# Functional and Taxonomic Structuring of Ground‐Dwelling Spider Communities Under the Combined Effects of the Environment and Metal Contamination

**DOI:** 10.1002/ece3.73958

**Published:** 2026-08-18

**Authors:** Aliénor Duval, Vincent Laderriere, Margot Jans, Jonathan Bouquerel, Philippe Usseglio‐Polatera, Aurélie Cebron, Florence Maunoury‐Danger

**Affiliations:** ^1^ Université de Lorraine, CNRS UMR 7360, LIEC Metz France; ^2^ INRAE Bordeaux Sciences Agro, ISVV, UMR 1065 SAVE Villenave‐d'Ornon France; ^3^ Université de Lorraine, CNRS UMR 7360, LIEC Nancy France

## Abstract

Northern regions of France have historically experienced mining and steel production activities, leading to the emergence of multi‐contaminated soils by metals. These metals are a source of indirect disruption to ecosystem functioning through the alteration of biodiversity. We focused on spiders, which are major regulators of terrestrial ecosystems, acting simultaneously as prey, predators and competitors in soil communities. The aim of this study was to assess the impact of metal contamination, among a set of environmental parameters, on the taxonomic and functional structures of spider communities; to identify indicator spider species; and to define trait syndromes specific to metal‐contaminated wastelands. We sampled spiders in 40 sites that represented a metal‐contamination gradient. We have shown that certain environmental parameters largely account for the taxonomic and functional structure of spider assemblages (e.g., soil granulometry, vegetation, pH). The concentrations of metals in the soil (Ag, Al, As, Cd, Mn, Pb, Sb and Zn) also had a significant but less pronounced impact. We have identified *Xerolycosa nemoralis* (Lycosidae) as an interesting indicator of metal‐contaminated environments. Furthermore, the contaminated sites contained fewer indicator species than the reference areas. Forested and herbaceous landscape promoted the presence of sheet‐ and orb‐web weavers, respectively. Although metal contamination was not the only driver of this trend, stenophagous spiders, which prefer to retreat and hunt on the ground, were selected at most contaminated sites surrounded by sealed ground, which corresponded to a selection of A‐strategists. The observed trends could be explained by the age of the contamination, the low bioavailability of the metals, the spiders' dispersal capacity, or their effective adaptative mechanisms that mitigate the direct impact of soil contamination on spider assemblages. A better understanding of the role of metals in shaping biological communities can provide valuable insights, particularly regarding ecosystem functioning, environmental monitoring and restoration of contaminated areas.

## Graphical abstract

Through a field study along a metal contamination gradient, we propose a combined application of taxonomic and functional approaches to study the impacts of contamination on polyphagous predators, spiders. We have demonstrated a significant impact of metal contamination on spider communities, although it operates among a set of variables (e.g., vegetation, soil granulometry) and identified indicator species and traits‐syndrome related to contamination. Our study provides new insight into the use of spiders as bioindicators of metal‐contaminated environments.

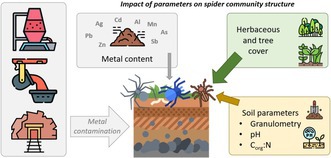

## 1 Introduction

Metals are naturally present in environmental matrices due to bedrock evolution, corresponding to a natural geochemical background (Baize 2009). Anthropogenic activities such as mining and steel processing industries, which are among the main sources of chronic and historical metal pollution (O'Connell et al. 2008), can cause an increase of their concentration in soils. Released metals can affect ecosystem functioning by altering the dynamics of biological communities, making them relevant pollutants to study (Pereira et al. 2018). For instance, metal contamination has been shown to alter soil invertebrate diversity. In soils, zinc (Zn) and copper (Cu) significantly decrease abundance of earthworm populations (at 2000 and 176 μg/g of soil respectively; Creamer et al. 2008; Nahmani and Lavelle 2002). At the surface, zinc (1–4 mg/g of soil) and cadmium (Cd; 59 ng/g of soil) reduce population density and alter the taxonomic composition of ground‐dwelling beetle communities by eliminating the Geotrupidae, Carabidae and Silphidae families (Osman et al. 2015; Skalski et al. 2015). Eeva et al. (2004) and Grześ (2009) highlighted how, in a multi‐metal contamination context, the specific composition of ant populations is modified in smelters and mining pits. Furthermore, in a former uranium mining area, although the abundance of some taxa (Clubionidae and Staphylinidae) was negatively correlated with soil concentrations of certain metals (e.g., Beryllium (Be) for Clubionidae; Al and Pb for Staphylinidae), no evidence of a significant impact of metal contamination on the global structure of the soil arthropod community was found (Antunes et al. 2012).

Metal contamination can alter not only the taxonomic structure but also the functional structure of invertebrate communities. Functional diversity can be studied through the use of species' functional traits, which have been defined as “any morphological, physiological, phenological and behavioral characteristics measurable at the individual level, from the cell to the whole‐organism, which can be considered as surrogates for the performance of an organism” (Pey et al. 2014; Violle et al. 2007). Functional traits of communities offer insight into the way ecosystems function. For example, by examining trophic regimes, Skalski et al. (2015) highlighted the potential impact of metals on litter decomposition (i.e., a key process in soil ecosystems) through a decrease in the abundance of detritivorous taxa. By extension, the entire food web stability can be compromised by a bottom‐up effect of prey abundance (i.e., detritivores) on predators (Kędzior et al. 2020). In addition, an increase in anthropogenic pressures is often linked to the functional homogenization of communities (i.e., becoming more functionally similar; Walker 1992). The potential negative effects of the latter can be mitigated if communities exhibit functional redundancy, that is, if several taxa share similar trait profiles, some of them can compensate for the loss of others (Lawton and Brown 1994). As an example of such a mechanism, Mori et al. (2015) showed that assemblages of oribatid mite living in litter, exposed to agricultural pressure resulting from monoculture in a larch plantations, exhibited a distinctive structure that limitated trait similarity, thereby mitigating functional homogenization. Functional homogenization has not yet been properly studied in the context of metal contamination, but it could be of interest for better understanding how ecosystem functioning can be disrupted by metal contamination and to potentially implement biotic remediation policies, as suggested by Walker (1992). Functional traits may also be linked to the evolutionary strategies of species within communities. Species adopting a K‐strategy tend to be associated with stable, unconstrained ecosystems and would exhibit, as an example, long life span, high investment in defense or parental care traits. On the other hand, those adopting an r‐strategy are more likely to survive in unstable ecosystems subject to disturbance, which is allowed by traits such as fast rate of development or high fecundity, making them pioneer species (MacArthur and Wilson 1967; Pianka 1970). When ecosystems are subject to strong but stable pressure (e.g., historical metal contamination of soil), species may share traits of both r‐strategists and K‐strategists, enabling them to survive in constrained environments. These species are known as ‘A‐strategists’ (Pianka 1970). The r‐K continuum has already been used to describe or compare the strategies adopted by invertebrates in response to metal contamination (Rusek and Marshall 2025; Sochova et al. 2006), but the strategy selection system used rarely refers explicitly to A‐strategists.

In the present study, we focused on spiders, which are among the most abundant and diversified group of epigeal polyphagous predators in soil macro‐invertebrate communities (Cardoso et al. 2011; Hendrickx et al. 2004). Spiders interact at various levels within the ecosystems they inhabit. They act as predators and ensure top‐down regulation of their prey populations. They serve as prey for birds, mammals, amphibians and reptiles, thereby ensuring bottom‐up regulation of these predator populations (Foelix 2010; Nentwig 1982). They also compete for resources with other ground‐dwelling predators, such as beetles (e.g., Staphylinidae and Carabidae; Nentwig 1982; Żmudzki and Laskowski 2012). Spiders also exhibit various mechanisms that enable them to survive, grow and reproduce in metal contaminated environments: for example, their high bioaccumulation capacities (Aziz et al. 2020), efficient excretion abilities (Hopkin 1989) or biochemical detoxification strategies (Babczyńska et al. 2006). The few existing studies indicate that metals do not seem to have a significant impact on taxonomic diversity but that they do play a role in modifying the composition of spider assemblages (Jung et al. 2008; Mannu et al. 2020; Żmudzki and Laskowski 2012). These studies carried out on a limited number of sites did not take into account their respective physicochemical characteristics, which can influence community response through variations in metal bioavailability in soils and variations in other soil characteristics such as texture, pH or Cation Exchange Capacity (CEC; Hendrickx et al. 2004). Furthermore, spiders' functional traits are still largely understudied, with the exception of a few studies related to vegetation structure or pest‐control in an agricultural context (e.g., Gallé et al. 2020; Öberg et al. 2007). The few studies carried out in the context of metal contamination have focused on a small number of model spider species and were conducted under laboratory conditions, studying the effect of a single metal at a time (Aziz et al. 2020; Babczyńska et al. 2013). Extending these studies to a multi‐metal contamination gradient at multiple sites would help us to evaluate the relevance of spiders as a biological model for studying the effects of environmental stressors on ecosystem functioning.

To address these research gaps, our study aimed to assess the extent to which the taxonomic and functional structures of spider assemblages can be affected by contamination from multiple metals. To this end, we studied a wide range of industrial sites, combining analyses of (i) site characteristics in terms of vegetation cover, (ii) soil physical and chemical characteristics, (iii) the abundance of spiders determined on the basis of morphological criteria, and (iv) associated functional traits. We hypothesized that the taxonomic composition of spider assemblages would be altered at metal contaminated sites compared to reference sites (hypothesis 1). We expected that the decline or disappearance of certain pollution‐sensitive taxa would be offset by the increase—or even emergence—of new pollution‐tolerant taxa, and therefore that there would be no significant changes in the level of taxonomic diversity within these assemblages (hypothesis 2). We assumed that these changes should be accompanied by modifications in the functional structure of assemblages linked to the selection of trait modalities enabling individuals to survive more effectively in a context of metal contamination (hypothesis 3). These changes could be associated with functional homogenization, which could reflect a change in the way metal‐contaminated ecosystems function (hypothesis 4).

## 2 Material and Methods

### 2.1 Study Sites, Sampling and Physicochemical Analyses

**FIGURE 1:**
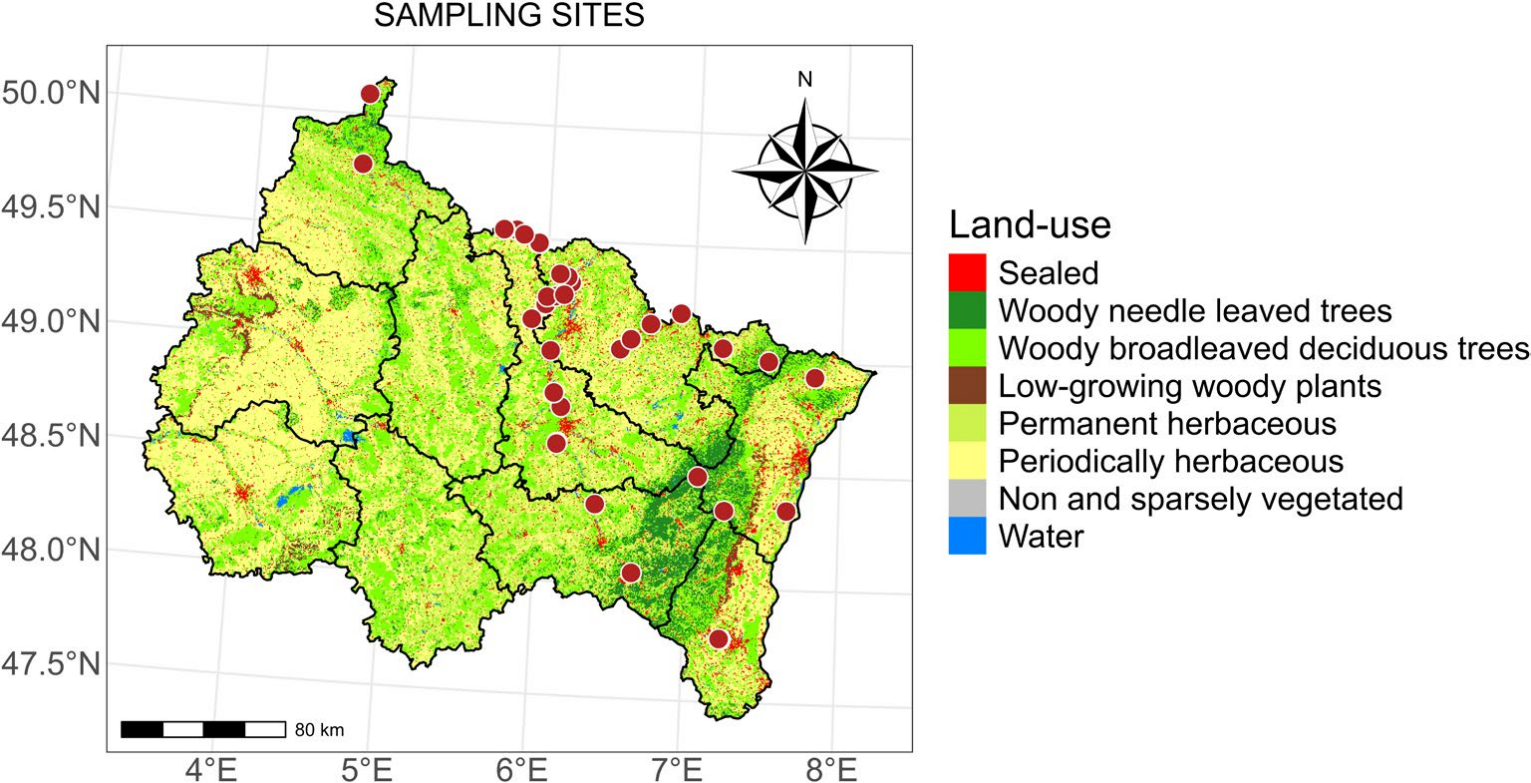
Presentation of the sampling sites in the Grand‐Est region of France. The red dots correspond to the location of the 40 sampling sites. The black lines correspond to the departmental boundaries. Each color corresponds to a land‐cover category defined by the Corine Land Cover (+) plus Backbone v.2018 with a resolution of 10 m.

We selected 40 sites in the Grand‐Est region of France (Figure 1) with contrasted levels of metal contamination. Sampling was carried out over two consecutive years during May to July (19 and 21 sites in 2022 and 2023, respectively). On each site, three 16 m^2^ subsites were defined at least 20 m apart from each other (40 sites × 3 subsites = 120 subsites, Figure S1) and were considered as replicates.

Tree and herbaceous vegetation covers were measured using one hemispherical picture and one ground quadrat picture (50 × 50 cm), respectively (Figure S1). These pictures were analyzed using the ImageJ software (Schneider et al. 2012) in order to calculate the percentage of green pixels, which was then used as a proxy of vegetation cover. An estimate of the primary production was assessed by collecting the plant biomass in two other quadrats (25 × 25 cm), and by measuring its dry weight in the laboratory. On each subsite, three soil samples (~500 g each) from the first top 30 cm were collected in order to obtain a representative composite sample. Unless otherwise stated, all subsequent analyses were performed on homogenized 2 mm sieved soil. The pH was measured on resuspended soil in distilled water (1:5 w/v; NF‐ISO‐10390 standard). Total N, C_org_, CaCO_3_, and CEC were measured (using NF‐ISO‐13878, NF‐ISO‐10694, NF‐EN‐ISO‐10693, NF‐X31‐130 and the Metson method, respectively). Soil texture was also determined by measuring the three main fractions (< 2 μm for clay, 2–63 μm for silt and 63–2000 μm for sand fraction; NF‐ISO‐11277). PAHs were extracted from 2 g sifted (200 μm) and dried soil (40°C), using pressurized‐liquid extraction in accordance with standard XP‐X33‐012 (50% acetone, 25% dichloromethane, 25% hexane; 150°C and 104 bars; Speedextractor E‐916, Büchi). The concentrations of the 16 US‐EPA (U.S. Environmental Protection Agency) PAHs were measured, as described in Cennerazzo et al. (2017), after accelerated solvent extraction (ASE 200, Dionex) using dichloromethane by reverse‐phase chromatography (UHPLC Ultimate 3000 system, Dionex) equipped with Diode Array Detector for UV detection according to NF‐EN‐17503. Finally, the elemental composition (total and extractable) of the soils was measured from 200 μg soil after digestion (0.1 M HNO_3_ and 0.01 M CaCl_2_) by using inductively coupled plasma spectroscopy (iCAP‐TQ‐ICP‐MS, Thermo Scientific). Procedural blanks were performed every 10 samples with milliQ water. In total, 17 elements were analyzed: Na, Mg, Al, P, K, Ca, Cr, Mn, Fe Co, Ni, Cu, Zn, As, Mo, Cd, and Pb. Concentrations of each metal and PAH, and corresponding quantification and detection limits are given in Table S1.

To account for the potential impact of the landscape on spider assemblages, the relative area of different land‐cover types within a radius of 5, 50 and 500 m around each subsite was determined using Corine Land Cover plus Backbone v. 2018 (European Comission, Copernicus Land Monitoring Service, and European Environment Agency 2023; available at: https://doi.org/10.2909/cd534ebf‐f553‐42f0‐9ac1‐62c1dc36d32c). Eight categories of land‐cover were present around the 120 subsites: sealed soils, woody needle or broadleaved deciduous trees, low‐growing woody plants, permanent or periodically herbaceous, non and sparsely vegetated, and water (see Figures S2–S4). Then, a first Principal Component Analysis (PCA) based on the land‐cover categories found at each radius for the 120 subsites was performed. The coordinates of the subsites on the first two axes of this PCA (broken stick model; Legendre and Legendre 1998) were used as landscape‐scale synthetic descriptors for these subsites. Subsequently, these synthetic landscape descriptors were not directly included as “active variables” in the multivariate analyses, but were added retrospectively as “additional variables” to simulate the potential landscape‐scale effects without blurring the primary effects of the local environmental parameters we measured.

### 2.2 Clustering of Sites

To account for the co‐occurrence of metals in the soil, we identified groups of co‐occurring metals based on their proximity (i.e., the similarity of their coordinates on successive factorial axes) in a second PCA which considered their respective concentrations in the soil (Table S1). This second PCA was performed based on the metal concentrations measured in the soils at the 120 subsites of the sampling design. A Hierarchical Clustering Analysis (HCA) was subsequently performed (using Euclidean distance and the “Ward.D2” linkage algorithm) based on the correlation coefficients between metal concentrations in the soils of the 120 subsites and the coordinates of these subsites along the five first PCA axes (broken stick model). This HCA allowed to identify six groups of co‐occurring metals: [Al_Mn], [Fe_Co_Ni_Cu], [Cr_Mo], [As_Sb], [Ag_Cd] and [Zn_Pb]. For each group of metals, the raw metal concentrations were combined into a Metal Index (MI; Lemmel et al. 2019) using the following formula:

$${\mathrm{MI}}_{k,j}=\sum_{i=1}^{i=n}\frac{{\left[{\text{metal}}_{i}\right]}_{\text{soil}\left(j\right)}\times 100}{{\left[{\text{metal}}_{i}\right]}_{\max}}$$

with: MI_k,j_: the metal index for the soil “j” and the group of metals “k”, n: the number of metals in the kth group, [metal_i_]_soil(j)_: the concentration of the metal “i” of the group “k” in the soil “j”, and [metal_i_]_max_: the maximal concentration of the metal “i” of the group “k” over the 40 sites. The values of the different MIs, available in the Table S1, were used in the following analyses in place of the raw concentrations.

A third PCA was performed based on metal‐related parameters (i.e., MI values for the groups of metals previously identified in the second PCA), plant cover (dry weight of plant biomass, tree and herbaceous cover), granulometry (sand and clay fractions) and other soil characteristics (pH, CEC, CaCO_3_, P, C_org_/N ratio and PAH content) in each of the 120 subsites. Synthetic land‐cover variables were added retrospectively as “additional variables”. To avoid having three replicates of the same site in different clusters, we performed a second HCA (based on Euclidean distance and Ward linkage algorithm) using the average coordinates (i.e., the barycenter) of the three subsites composing each of the 40 sites on the first two PCA axes selected by the broken stick model. Six clusters of sites (A–F) were identified, allowing for the grouping of sites with similar metal contamination profiles as well as other similar environmental parameters. The letters were assigned to represent an increasing gradient of MIs from cluster A to cluster F (Figure S5). Since all environmental variables were taken into account when clustering the sites, these clusters should be interpreted in terms of the overall environmental conditions of the sites and not solely in terms of soil metal contamination.

### 2.3 Spider Sampling

Spiders were sampled using pitfall traps left in the field for 7 days (4 traps × 3 subsites × 40 sites = 480 samples) a quarter filled with 1,2‐Propandiol (≥ 99.5%, Ph. Eur.; Carl Roth). Individuals were collected from pitfall traps and preserved in jars filled with ethanol (96%; VWR). They were identified using morphological criteria and identification keys to the finest taxonomic level possible (mainly species level; Nentwig et al. 2010; Oger 2011; Roberts 2020). Juveniles and damaged individuals were redistributed in proportion to those identified at lower taxonomic levels (genus and species) for each subsite to best reflect the abundance of sampled taxa.

### 2.4 Spider Functional Traits

A trait database has been created to study the functional responses of spider communities. This database includes 32 traits for 219 taxa (all taxonomic levels considered) from 26 spider families. The biological information already available was aggregated from the BETSI (Joimel et al. 2021, downloaded on October 2022), World Spider Trait (Pekár et al. 2021, downloaded on October 2023), ARAMOB (Bach et al. 2024, visited in February 2024), and Spiders of Europe (Nentwig et al. 2010, visited in November 2023) databases. This information was supplemented by an analysis of the available scientific literature on spider traits, considering 71 scientific articles. In addition, four other databases, 13 books about the biology of spiders, and six web sites were used. All references are listed in Data S1. Each functional trait was described with different modalities. Modalities refer to the different values, states, or forms that a trait can take. For example, the trait “maternal care” is described by four modalities: “none”, “eggsac guarding”, “spiderlings guarding”, and “spiderlings feeding”. All the modalities of the studied traits are presented in Table 1. The information available was coded using the fuzzy coding method (Chevenet et al. 1994), which aims to assign to each taxon an affinity score for each of the different modalities describing a given trait (from 0 = no affinity to 3 = high affinity). The set of affinity scores assigned to each “taxon × trait” pair was then converted into a distribution of relative frequencies, with the sum of the taxon's scores for a given trait always being equal to 1. At the community level, these trait profiles, weighted by the log‐transformed abundances of the corresponding taxa in the community, were averaged for each subsite (Community Weighted Mean method; Lepš and de Bello 2023).

**TABLE 1:** Definition of the spider traits, their modalities and abbreviations.

Trait	Definition of traits	Modalities	Abbreviation	Definition of modalities
Active Period	Number of months spiders are active Spiders are active for mating, hunting and are not in hibernation	6 months or less*	<_6_months	Active up to 6 months per year (*groups active less than 2 months and 3 to 6 months modalities)
		Between 7 and 11 months	7_to_11_months	Active seven to eleven months per year
		All year long	All_year	Active all year long
Maternal care	Care provided by the female to her eggs or offsprings Based on modalities found in World Spider Trait Database (Pekár et al. 2021)	Eggsac guarding	Egg_guard	Female looks after the eggsac by keeping it in her web, carrying it with her or watching from a distance
		None	No_care	Female does not care of eggs or offsprings
		Spiderlings guarding	Spiderling_guard	Female looks after the offsprings by keeping them in her web, carrying them on her back or watching from a distance
		Spiderlings feeding	Spiderling_feed	Female feeds her offsprings; it includes laying trophic eggs, prey sharing, regurgitation and matriphagy
Diet	Diet breadth Description by Pekár et al. (2011)	Euryphagous	Euryphagous	Spiders that do not have a specialized diet and can feed on many different types of prey
		Stenophagous	Stenophagous	Spiders whose diet specializes on a single type of prey or a small number of prey types
Prey type	Taxonomy of spider's prey Based on modalities found in World Spider Trait Database (Pekár et al. 2021)	Araneae and other arachnids	Arach	Spiders or other arachnids (e.g., opiliones)
		Coleoptera	Coleo	Coleopterans
		Collembola	Coll	Springtails
		Dermaptera and Blattodea	Derma_Blatt	Dermapterans (e.g., forficula) or Blattodea (e.g., cockroaches or termites)
		Diptera	Dip	Dipterans (e.g., flies)
		Hemiptera	Hemi	Hemipterans (e.g., cicadas, aphids, leafhoppers)
		Hymenoptera	Hymeno	Hymenopterans (e.g., ants, wasps, bees)
		Isopoda	Iso	Isopoda (e.g., woodlice)
		Lepidoptera	Lepi	Lepidopterans (e.g., butterflies, moths)
		Orthoptera	Ortho	Orthopterans (e.g., grasshoppers, crickets)
Hunting Guild	Ecological hunting guild Based on modalities found in World Spider Trait Database (Pekár et al. 2021); Description of guilds by Cardoso et al. 2011	Cursorial hunter	Cursorial	Active hunting method: spiders are fast‐moving and specialize in the rapid pursuit of their prey
		Ground hunter	Ground	Active hunting method: spiders only hunt prey on the ground
		Sheet web weaver	Sheet_web	Passive hunting method: the spiders wait on a hammock‐shaped web surrounded by a few lines of web. Flying prey strikes these lines and falls into the hammock area. They are often found in shrubs, trees, low vegetation or depressions in the ground
		Space web weaver	Space_web	Passive hunting method: spiders build a 3D web, often messy, to trap prey that falls into it
		Other hunter*	Other_hunt	All other hunting strategies (*groups orb web weaver, sensing hunter, stalker and ambusher modalities)
Web type	Type of web build	Sheet web	Sheet	Build sheet web, corresponding to tridimensional web with a net into which prey fall
		Space web	Space	Build a complex tridimensional web in which prey become entangled
		Orb web	Orb	Build vertical plane web composed of rays connected by spiral support wires
		Tube	Tube	Build tubes entirely lined with silk
		No web	No_web	Spider that does not construct web (wanderer spiders)
Retreat	Location of the retreat used to avoid predation, to rest or to mate Based on modalities found in World Spider Trait Database (Pekár et al. 2021)	Among stones	Stones	Retreat located among stones
		In soil	In_soil	Retreat located in the soil (e.g., in a burrow)
		In vegetation	Vegetation	Retreat located in the vegetation (e.g., in leaf)
		No retreat	No_retreat	No retreat built
		On the ground	On_soil	Retreat located on soil surface
		On web	On_web	Retreat located on the spider's web
		Under bark	Under_bark	Retreat located under bark (of dead or living trees)

*Note:* The symbol * indicates that relative frequencies of several modalities (used less than 1%) have been grouped together, in which case the modalities are indicated. See Table S1 for fuzzy coding of each trait.

The seven best‐documented traits (> 50% of taxa described) for the taxa included in subsequent analyses (see the dataset filtering described in data analyses section) were selected. For a given trait, modalities with an average relative frequency of less than 1% across all sampled spider communities were grouped together. Selected traits relate to habitat (retreat), reproduction (maternal care), predation (hunting guild, diet, prey type), web construction (web type) and life cycle (active period). Those seven traits represent a total of 36 different modalities (Table 1). Fuzzy coded profiles of spiders are available in Table S2.

### 2.5 Data Analyses

All subsequent statistical analyses were performed using R software, v.4.3.3 (R Core Team 2025). They were performed at the subsite scale (*n* = 120). The subsite distribution in PCA and the contribution of the environmental parameters to the latter were visualized using *FactoMineR* (v.2.10; Husson et al. 2024) and *factoextra* (v.1.0.7; Kassambara and Mundt 2020) packages. Individuals from the four pitfall traps from one subsite were pooled. After redistribution of some individuals as explained in the “spider sampling” section, the taxonomic dataset was rarefied by removing very rare taxa. To do this, families with fewer than three individuals or appearing in fewer than three subsites were removed from the dataset, in order to eliminate any potential non‐ground dwelling spiders. To visualize the overall taxonomic structure of spider communities, total abundance and three taxonomic indices were calculated using the *vegan* package (v.2.6‐4; Oksanen et al. 2024): taxonomic richness, Shannon‐Weaver diversity, and Pielou evenness. Functional diversity was described by calculating three indices using the *FD* package (v.1.0‐12.3; Laliberté et al. 2023; Villéger et al. 2008): functional divergence, richness and evenness. Next, in order to identify significant variations in land‐cover or in taxonomic and functional metrics, depending on the level of metal‐contamination and/or variations in other environmental parameters, the six clusters of sites were compared by performing a Kruskal–Wallis test followed by a *post hoc* Dunn test with Bonferroni *p*‐value adjustment. When significant differences were observed between clusters, Generalized Additive Models (GAM) were developed, which enabled to identify the set of environmental parameters that drove significant variations in metric values, whether they had a linear or non‐linear relationship, with the calculated metrics. The indicator species and indicator trait modalities for each cluster were identified using the “IndVal.g” algorithm (999 permutations), which includes correction for unequal group sizes by using the *indicspecies* package (v.1.7.14; Cáceres et al. 2024; Cáceres and Legendre 2009). As recommended in Cáceres and Legendre (2009), a Benjamini‐Hochberg (BH) correction of the *p*‐value was chosen, which allows to limit the false discovery rate in order to be more confident in reporting species as indicators (*p*‐value < 0.05). A Canonical Correspondence Analysis (CCA) was applied to the log‐transformed species abundance table to explain taxonomic differences observed in spider communities in response to variation in the 17 environmental descriptors. To statistically validate the results, a permutational multivariate analysis of variance (PERMANOVA, 999 permutations) was conducted to test the significance of (i) the CCA itself, (ii) each CCA axis, and (iii) the explanatory power of each parameter (*p*‐value < 0.05). The same procedure was used to link the mean trait profiles of spider assemblages to the environmental parameters.

## 3 Results

### 3.1 Soil and Land‐Cover Characterization

**FIGURE 2:**
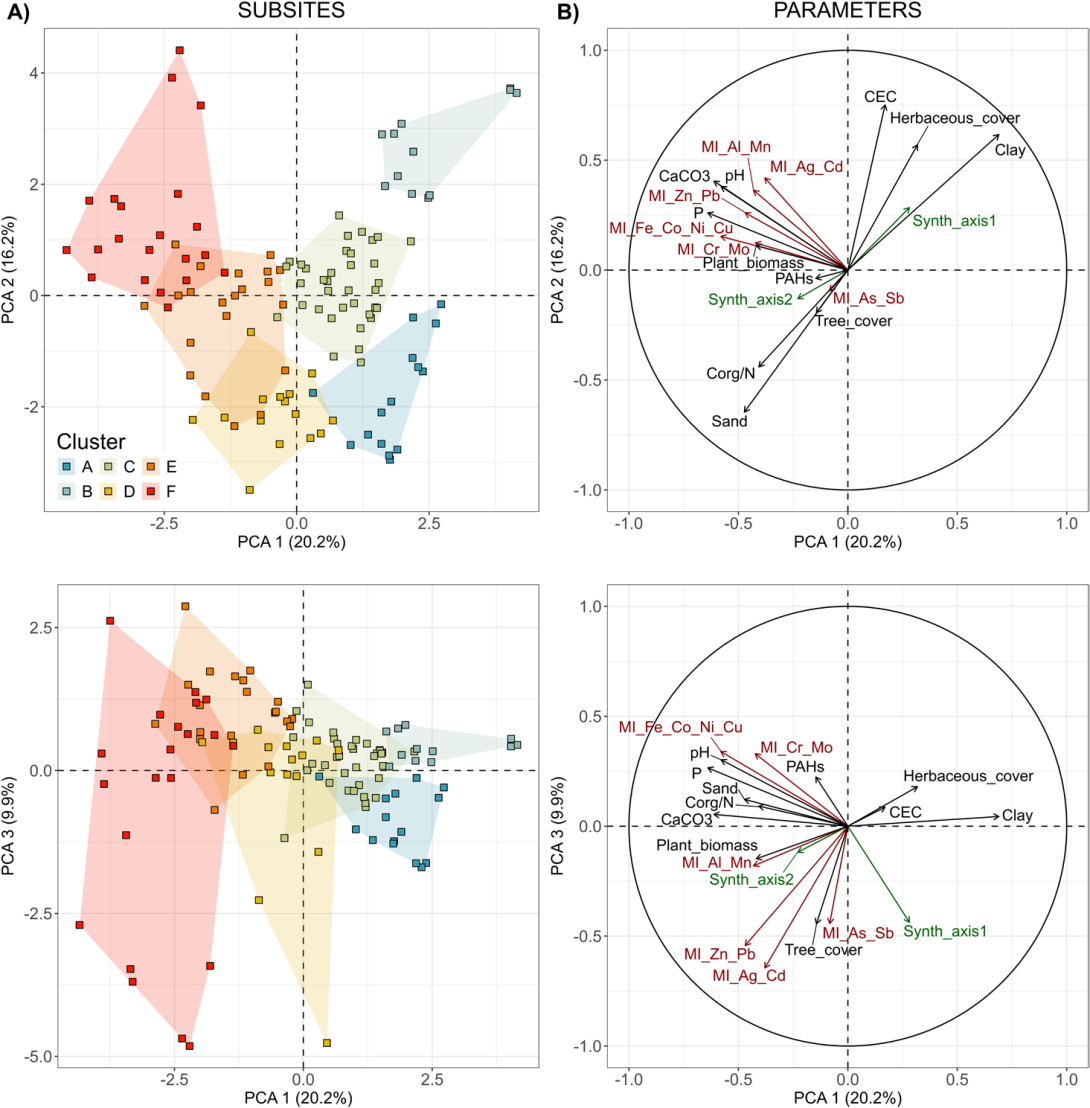
Results of the PCA analysis of the 120 subsites based and their environmental parameters. Distribution of the subsites belonging to the six clusters of sites (clusters A to F) in the factorial planes PCA1‐PCA2 (A) and PCA1‐PCA3 (C), and linear correlations between environmental parameters and subsite coordinates along PCA1 (B, D), PCA2 (B) and PCA3 (D). The green arrows correspond to synthetic landscape variables (see Material and Methods section and Figure S6). These variables were not included in the PCA analysis but were added to the graphs as supplementary variables, after the correlation coefficients between these variables and the principal components has been calculated. The red arrows correspond to metal‐related parameters. The boundaries of a cluster correspond to the convex polygon that encloses all the subsites of that cluster.

**FIGURE 3:**
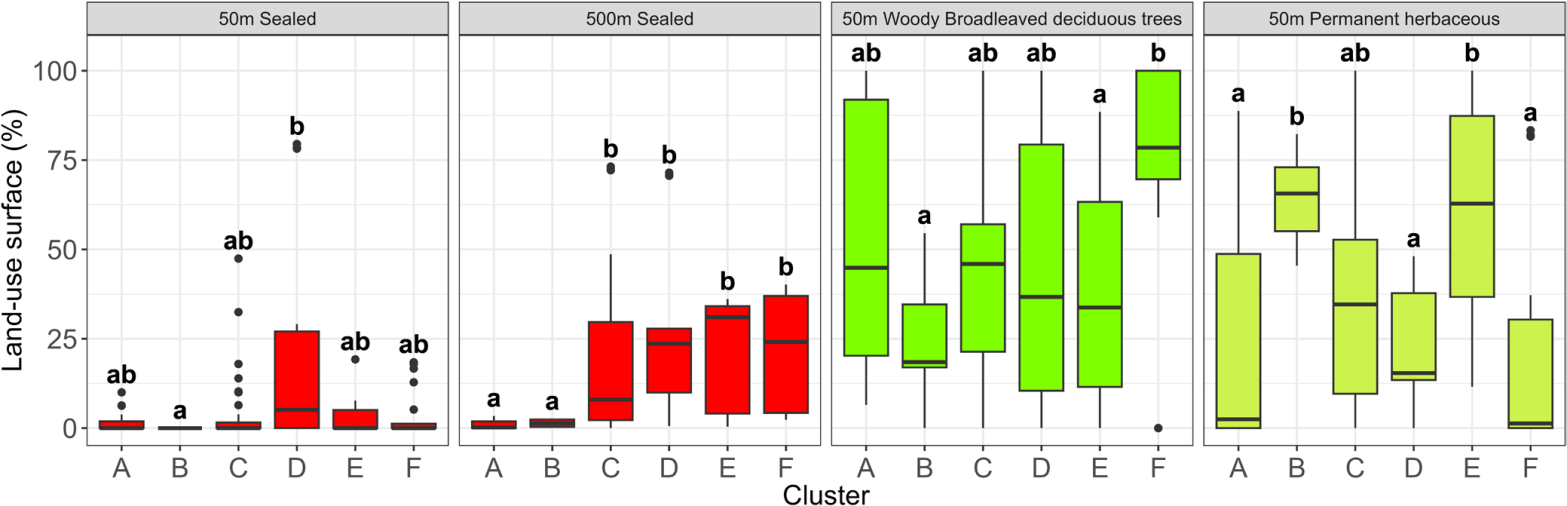
Surface percentage of the most important land‐use categories in the six clusters of sites (see Figures S6 and S7). Significant differences were assessed using Kruskal–Wallis tests, followed by Dunn *post hoc* tests with Bonferroni *p*‐value adjustment. Different letters indicate clusters with significant differences for a given index (adjusted *p*‐value < 0.05).

Based on the third PCA analyzing environmental and metal‐related parameters (i.e., MI values for the groups of metal previously identified) among the 120 subsites, 46.4% of the total variance was explained by the first three axes (Figure 2). Subsites were located along a gradient of metal contamination, mainly represented along the first axis, which increases from cluster A (PCA 1 > 0) to cluster F (PCA 1 < 0), with rather similar levels of soil metal content for clusters B, C and D (Figure S5). However, these clusters exhibited marked differences in herbaceous cover (maximum in B), granulometry (maximum sand content in D) and C_org_/N ratio (also maximum in D). Three subsites of cluster F, located on ancient blast furnace settling ponds, presented the strongest positive coordinates along PCA 2 due to very high values of MI_Ag_Cd (Figure 2A,B). This metal index, as well as MI_Zn_Pb, was the parameter most negatively correlated with PCA 3 (Figure 2D). Among the other environmental parameters, pH, CaCO_3_ and P content were highly contributive and negatively correlated to PCA 1, as was the MI_Fe_Co_Ni_Cu (Figure 2B and Figure S6). PAH content was among the least contributive variables despite cumulated concentrations ranging from 0.08 to 1050.8 μg/g among subsites (< 3% for each factorial plane, Figure S6). Furthermore, the subsites' soils of clusters C to F were significantly more sealed than those of clusters A and B, especially within a 500‐m radius around the subsites (Figure 3). This “soil sealing” gradient was taken into account by the second landscape‐based synthetic descriptor (Synth_axis2; see Figures S7 and S8). However, this synthetic variable, negatively correlated with PCA 1 and PCA 2 (−0.22 and −0.13, respectively; Figure 2), was much less correlated with PCA 1 than many other environmental variables. In contrast, herbaceous cover, CEC and clay content, as well as the first landscape descriptor (Synth_axis1, which described a “forest to herbaceous” land‐cover gradient; Figures S7 and S8), were positively correlated with both PCA 1 and PCA 2, and were strongly associated with subsites of cluster B (Figure 2B and Figure S5). This cluster did indeed exhibit a high proportion of permanent herbaceous cover within a 50‐m radius around the subsites (Figure 3 and Figure S3), a proportion higher than that observed around the subsites of cluster A (*p*‐value < 0.01) which had a high tree cover (Figure S5) within a 50 and 500‐m radius (Figure 3; “needle leaved trees” and “broadleaved deciduous trees” in Figures S3 and S4). Therefore, based on vegetation cover, cluster A and cluster B can be considered as woodland and grassland references, respectively.

### 3.2 Abundance and Taxonomic Diversity of Spider Assemblages

**FIGURE 4:**
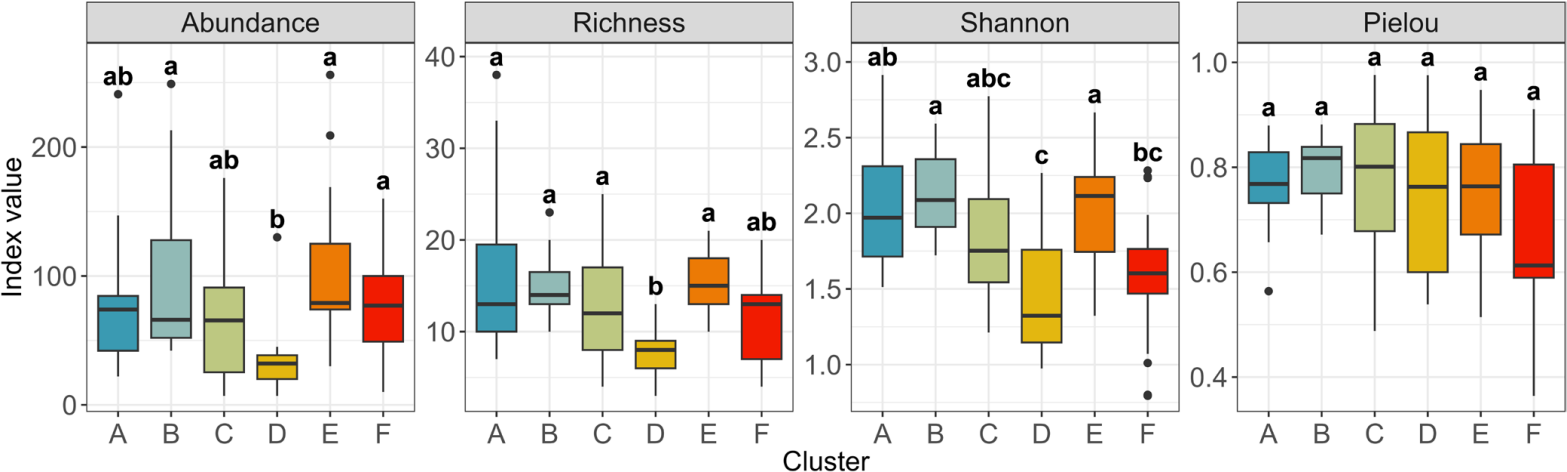
Taxonomic descriptors of spider assemblages in the six clusters of sites. Total abundance, taxonomic richness, Shannon–Weaver diversity index and Pielou evenness index variations are presented. Significant differences were assessed using Kruskal–Wallis tests, followed by Dunn post hoc tests with Bonferroni *p*‐value adjustment. Different letters indicate clusters with significant differences for a given index (adjusted *p*‐value < 0.05).

**FIGURE 5:**
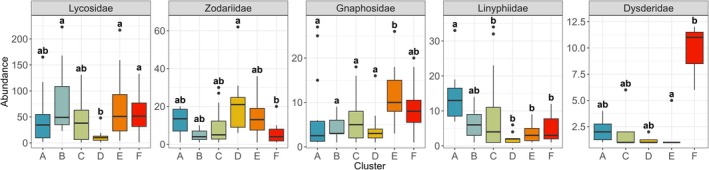
Abundance of the five families of spiders showing significant differences in taxonomic descriptors between the six clusters of sites. Significant differences were assessed using Kruskal–Wallis tests, followed by Dunn post hoc tests with Bonferroni *p*‐value adjustment. Different letters indicate clusters with significant differences for a given index (adjusted *p*‐value < 0.05).

A total of 8911 spiders belonging to 19 families, 89 genera and 155 species were collected. The Lycosidae family dominated the assemblages, accounting for 64% of captured individuals, followed by the Zodariidae (8.83%), Gnaphosidae (8.53%) and Linyphiidae families (7.46%). We highlighted significant variations in taxonomy‐based descriptors and the abundance of certain spider families among the different clusters (Figures 4 and 5). Generalized Additive Models (GAM) revealed that taxonomic metrics were influenced by various environmental parameters. The total abundance of spider assemblages was primarily influenced by herbaceous cover and pH (*p*‐values < 0.001; Figure S9), whereas taxonomic richness and diversity were more strongly influenced by metal‐related parameters such as MI_Ag_Cd (*p*‐value < 0.001 for both metrics) or MI_Zn_Pb (*p*‐value < 0.01 for richness; Figures S10 and S11). MI_Ag_Cd appears to reflect an important impact of these metals on the richness and diversity of spider assemblages, with a marked decline in richness and diversity at the beginning of the contamination gradient, followed by a phase of less pronounced decline (diversity) or even partial recovery (richness) at higher contamination levels. At the cluster scale, cluster D, characterized by a high sand content and a high C_org_/N ratio (Figure 2 and Figure S5), exhibited significantly lower abundance, richness and Shannon‐Weaver diversity than other clusters (Figure 4). Subsites within this cluster displayed the highest abundance of the Zodariidae family (composed of the two species *Zodarion italicum* and *Zodarion rubidium*), but the lowest abundance of the Lycosidae (Figure 5). Their abundance increased significantly in subsites with the highest herbaceous cover such as in the grassland reference sites of cluster B (*p*‐value < 0.001; Figure S12). Conversely, the forested reference sites of cluster A harbored a higher abundance of the Linyphiidae family (Figure 5). GAM showed that Linyphiidae abundance slightly decreased along the “forested to herbaceous” landscape gradient represented by Synth_axis1 (*p*‐value = 0.03; Figure S13), as well as with increasing MI_Fe_Co_Ni_Cu values (*p*‐value < 0.01). Additionally, cluster E exhibited a significantly higher abundance of the Gnaphosidae family, (primarily represented by *Drassyllus praeficus* ), than the first four clusters (Figure 5). Finally, cluster F exhibited significantly lower taxonomic diversity than clusters B and E (Figure 4) and had the highest abundance of the Dysderidae family (Figure 5).

### 3.3 Influence of Environmental Parameters on the Taxonomic Composition of Spider Assemblages

**FIGURE 6:**
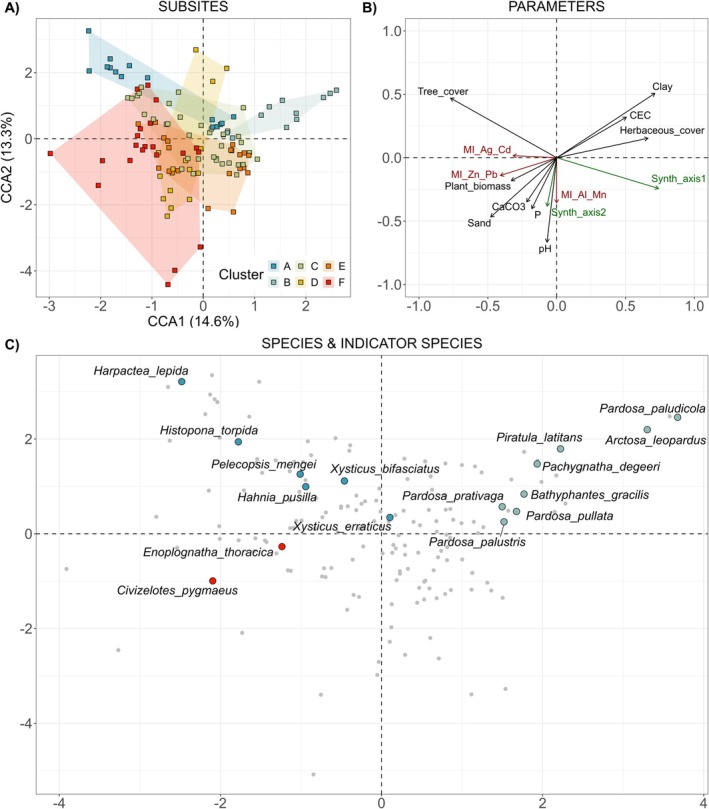
Canonical Correspondence Analysis (CCA) between spider abundances in subsites and environmental parameters. (A) Location of the 120 subsites and their clusters in the first factorial plane of the CCA. (B) Canonical weights of environmental parameters having a significant impact on species distribution (ANOVA‐like permutation test, *p*‐value < 0.05), with red arrows corresponding to metal‐related parameters and green arrows to synthetic landscape variables (see the legend in Figure 2 for more details). (C) Location of species indicative of a single cluster (large colored dots with species names) and other spider species (small gray dots) in the first factorial plane.

A Canonical Correspondence Analysis (CCA) was used to link species abundances to environmental parameters (Figure 6). A PERMANOVA revealed that the results obtained from the CCA were significant (*p*‐value = 0.001). CCA1 and CCA2, which together accounted for 27.9% of the variation in species abundances between subsites, were both significant (*p*‐value = 0.001). Twenty‐two percent of the variation in species abundances was constrained by environmental parameters. While three metal indices (MI_Zn_Pb, MI_Ag_Cd and MI_Al_Mn) exerted a significant effect, other local environmental parameters contributed more to shaping the spider communities (Figure 6B). Most of the subsites from clusters D, E and especially F had a negative coordinate on CCA1, which corresponded to the highest values of these MIs as well as high values of plant biomass, CaCO_3_, P concentrations and soil sand content. Additionally, high soil pH and high scores on Synth_axis2 (representing the “sealing” gradient) were negatively associated with the coordinates of the subsites on CCA2. Furthermore, tree cover, herbaceous cover and Synth_axis1 were strongly aligned with CCA1, reflecting a land‐cover gradient that ranged from broadleaved forests (associated with cluster A subsites on the negative side of CCA1) to permanent grasslands (associated with cluster B subsites on the positive side of CCA1).

On the other hand, based on this analysis, we have identified indicator species for each cluster (Table 2). More indicator species were found in reference clusters (A and B) than in other ones. The six indicator species in cluster A (Figure 6C) appeared to follow each other in order of increasing tree cover from *Xysticus erraticus* (Thomisidae) to *Harpactea lepida* (Dysderidae). Similarly, the eight indicator species for cluster B appeared to follow primarily an increasing gradient of herbaceous cover, CEC, and soil clay content in permanent grasslands, from *Pardosa palustris* to *Pardosa paludicola* . Contrastingly, the two indicator species in cluster F were the only indicator species negatively located along CCA1 and CCA2 (Figure 6C). No spider species specifically indicative of clusters C, D, and E were identified. Several taxa were significant indicators of several clusters of sites, such as *Tenuiphantes tenuis* and *Trochosa robusta*, which were indicative of the two reference clusters A and B (Table 2). *Xerolycosa nemoralis* was also identified as an indicator species for more contrasted clusters (D and F).

**TABLE 2:** Indicator species of the different clusters of sites. Identification based on a multi‐level pattern analysis using the “*IndVal.g*” algorithm and Benjamini‐Hochberg corrected *p*‐values.

Family	Genus	Species	Indval	Corrected *p*‐value	Indicator of cluster(s)					
					A	B	C	D	E	F
Agelenidae	*Histopona*	*Torpida*	0.667	0.016	X					
Dysderidae	*Harpactea*	*Lepida*	0.447	0.030	X					
Gnaphosidae	*Civizelotes*	*Pygmaeus*	0.488	0.030						X
Gnaphosidae	*Drassyllus*	*Praeficus*	0.684	0.025		X	X		X	X
Hahniidae	*Hahnia*	*Pusilla*	0.600	0.016	X					
Linyphiidae	*Bathyphantes*	*Gracilis*	0.478	0.019		X				
Linyphiidae	*Pelecopsis*	*Mengei*	0.618	0.016	X					
Linyphiidae	*Tenuiphantes*	*Flavipes*	0.563	0.036	X		X			X
Linyphiidae	*Tenuiphantes*	*Tenuis*	0.702	0.016	X	X				
Lycosidae	*Arctosa*	*Leopardus*	0.577	0.016		X				
Lycosidae	*Aulonia*	*Albimana*	0.801	0.019	X	X	X		X	X
Lycosidae	*Pardosa*	*Hortensis*	0.762	0.016			X	X	X	X
Lycosidae	*Pardosa*	*Paludicola*	0.500	0.016		X				
Lycosidae	*Pardosa*	*Palustris*	0.602	0.019		X				
Lycosidae	*Pardosa*	*Prativaga*	0.836	0.016		X				
Lycosidae	*Pardosa*	*Pullata*	0.891	0.016		X				
Lycosidae	*Pardosa*	*Saltans*	0.816	0.016	X		X		X	X
Lycosidae	*Piratula*	*Latitans*	0.440	0.030		X				
Lycosidae	*Trochosa*	*Robusta*	0.661	0.019	X	X				
Lycosidae	*Xerolycosa*	*Nemoralis*	0.580	0.019				X		X
Tetragnathidae	*Pachygnatha*	*Degeeri*	0.743	0.016		X				
Theridiidae	*Enoplognatha*	*Thoracica*	0.572	0.016						X
Theridiidae	*Euryopis*	*Flavomaculata*	0.559	0.019	X					X
Thomisidae	*NA*	*NA*	0.491	0.019		X		X		
Thomisidae	*Ozyptila*	*Simplex*	0.559	0.019		X			X	
Thomisidae	*Xysticus*	*Bifasciatus*	0.447	0.025	X					
Thomisidae	*Xysticus*	*Erraticus*	0.464	0.025	X					

### 3.4 Functional Structure of Spider Assemblages

**FIGURE 7:**
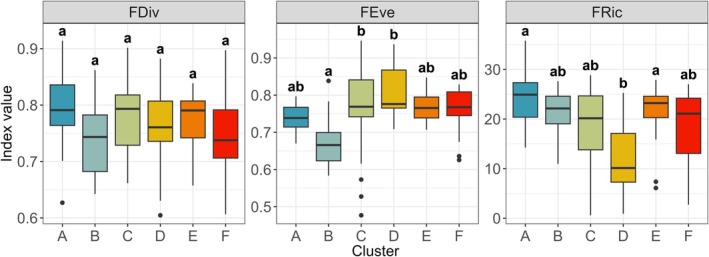
Functional metrics describing spider assemblages in the six clusters of sites. Variations in functional divergence (FDiv), functional evenness (FEve), and functional richness (FRic) are presented. Significant differences were assessed using Kruskal–Wallis tests, followed by Dunn post hoc tests with Bonferroni *p*‐value adjustment. Different letters indicate clusters with significant differences for a given functional metric (adjusted *p*‐value < 0.05).

Three functional indices were calculated and compared among clusters of sites (Figure 7). Functional divergence (FDiv) did not show any differences among clusters. Cluster B exhibited significantly lower functional evenness (FEve) than clusters C and D. Lastly, cluster D had a significantly lower functional richness (FRic) than clusters A and E.

### 3.5 Influence of Environmental Parameters on the Functional Trait Structure of Spider Assemblages

**FIGURE 8:**
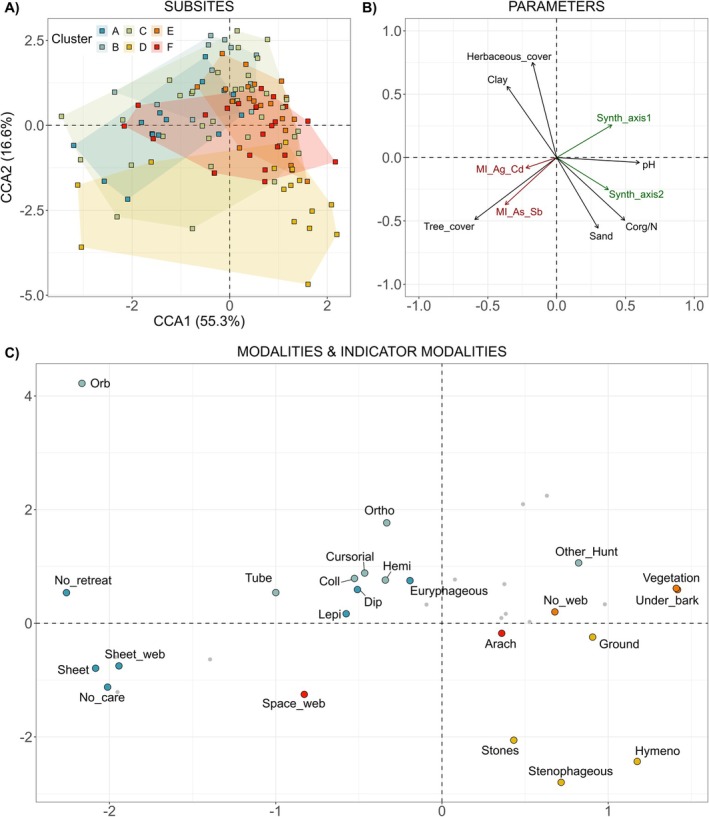
Canonical Correspondence Analysis (CCA) between the 36 modalities of 7 spider functional traits and environmental parameters. (A) Location of the 120 subsites and their clusters in the first factorial plane of the CCA. (B) Canonical weights of environmental parameters having a significant impact on species trait modality distribution (ANOVA‐like permutation test, *p*‐value < 0.05), with red arrows corresponding to metal‐related parameters and green arrows to synthetic landscape variables (see the legend in Figure 2 for more details). (C) Location of indicator trait modalities (large colored dots with trait modality acronyms) and other trait modalities (small gray dots) of seven traits in the first factorial plane. Detailed description of each trait and its modalities is given in Table 1.

A CCA was used to link mean trait profiles of spider assemblages to environmental parameters (Figure 8). Seven traits were studied, representing a total of 36 modalities. The PERMANOVA showed that the results of the CCA were significant (*p*‐value = 0.001). CCA1 and CCA2, which together accounted for 71.9% of the variation in trait profiles of spider assemblages between subsites, were both significant (*p*‐value = 0.001). In addition, 39.6% of the variation in trait profiles was constrained by environmental parameters. Two groups of metals (As_Sb and Ag_Cd) had a significant impact on the functional structure of spider communities (Figure 8B), although many other local parameters contributed more to this structure. PAHs did not have a significant impact on the trait structure of the spider assemblages (PERMANOVA; *p*‐value > 0.05). In addition, several indicator modalities of clusters E and F were positively located on CCA1 (Figure 8C), mainly driven by soil pH values and, to a lesser extent, by the two land‐use synthetic variables (e.g., retreat located under bark, no web building). We can note the particular location of the space web modality, which seems to be more closely linked to high tree cover and, to a lesser extent, to MI_As_Sb. Most of the indicator trait modalities of cluster D (e.g., stenophagous spiders, with retreat located among stones, and/or feeding on Hymenoptera) were related to high sand content in soil, high C_org_/N ratio, and quite high pH values in sealed soils (Figure 8B,C). High tree cover and high herbaceous cover were respectively associated with modalities indicative of forested sites in reference cluster A (e.g., spiders building and hunting with sheet webs, exhibiting no maternal care) and grassland sites in reference cluster B (e.g., spiders feeding on Orthoptera). The orb web trait modality, indicator of cluster B, clearly stood out from the others (CCA1 < 0 and CCA2 > 0). The active period was the only trait without indicator modality.

## 4 Discussion

### 4.1 Spiders' Taxonomic Assemblages Are More Influenced by Vegetation Cover and Soil Parameters Than by Metal Contamination

We hypothesized that metals would participate in modifying the taxonomic composition of spider assemblages (hypothesis 1), without significantly altering the taxonomic structure (hypothesis 2). Despite no clear tendencies among clusters, several significant differences were found according to taxonomic‐based descriptors of the entire spider assemblages and for five spider families (Figures 4 and 5). These variations appeared to be primarily related to land‐cover, vegetation cover and other soil characteristics (i.e., soil granulometry, CEC, pH, P and CaCO_3_ content) and, to a lesser extent, to metal‐contamination (as indicated by the MI_Ag_Cd, MI_Zn_Pb and MI_Al_Mn indices; Figure 6). Similarly, Mannu et al. (2020) showed that spider abundance and richness did not vary significantly as a function of Pb and Zn contamination in post‐mining areas. Żmudzki and Laskowski (2012) also found no clear variation in spider abundance and evenness along a gradient of Zn (from 54.3 to 2229.0 μg/g of soil, while in our study, Zn gradient extended from 9.4 to 95821.0 μg/g). They also showed that mesophilic meadows were generally richer than the harsher xerophilic meadows, the latter being comparable to the sites of cluster D (i.e., those with low herbaceous cover). Finally, Jung et al. (2008) observed no significant changes in taxonomic diversity under lower ranges of Pb (14.8–45.6 μg/g compared to 7.8–6949.0 μg/g in our study) and Cd contamination (0.3–1.4 μg/g compared to 0.03–105.6 μg/g in our study). Although the concentrations of Zn and Pb measured in our study frequently exceeded the Ecological Soil Screening Levels for invertebrates (Eco‐SSLs) established by US‐EPA (with 79 subsites exceeding 120 μg/g for Zn and 9 exceeding 1700 μg/g for Pb; US EPA 2015), we found only a minor impact of metal‐related parameters on the taxonomic structure of spider assemblages. Consequently, taxonomic structure indices (such as richness and diversity) do not appear to be the most sensitive indicators for assessing the potential alteration of spider assemblages by soil metal contamination. In terms of taxonomic composition, the species of the Lycosidae family were clearly associated to permanently herbaceous meadows with a few broadleaved deciduous trees within a 50 m radius (Figures 5 and 6). A high habitats diversity in a landscape, including grassland and wooded areas, positively influences the abundance and richness of this family, in accordance with previous observations (Öberg et al. 2007; Theron et al. 2020). On the other hand, metals still appeared to have a selective influence on the Lycosidae family (Figures 5 and 6; Table 2). The indicator species of this family were all negatively correlated with MI_Zn_Pb and MI_Ag_Cd (e.g., the four species of the genus *Pardosa*). Chen et al. (2011) reported slowed growth and a decrease in the number of eggs produced by *Pardosa astrigera* following the consumption of water contaminated with 100 mM Zn and Pb, suggesting that a greater proportion of energy was devoted to detoxification. In metal‐contaminated environments, certain species of Lycosidae may express particular survival strategies. More specifically, 85% of *X. nemoralis* individuals were recorded at subsites in clusters D and F, for which *X. nemoralis* serves as an indicator species (Table 2). *X. nemoralis* is known for laying fewer, but more energy‐rich eggs in a context of metal contamination. While these features increase the hatching rate, they also delay the hatching date in the context of contamination by Cu, Cd, Zn and Pb (Babczyńska et al. 2012, 2013). This strategy, which aims to maximize egg survival in a stable but constrained environment makes *X. nemoralis* a typical A‐strategist (Greenslade 1983) and an interesting indicator species for environments with high and stable metal contamination (as is the case for sites in clusters D and F). Cluster E showed contamination similar to those of clusters D and F for metals. However, and despite a lack of statistical significance, this group of subsites was characterized by lower extractable metal concentrations (Table S1) which could explain the lower abundance of this specific species in this cluster. It would be of interest to extend our knowledge on egg survival or egg energy content to other identified indicator species, in order to confirm this potential link with metal contamination.

Soil contamination by several metals also contributes to changes in the taxonomic composition of ant (Eeva et al. 2004; Grześ 2009) or beetles communities (Skalski et al. 2015). However, other natural environmental factors may also play a role and are too rarely studied in conjunction with anthropic contamination. In our dataset, spider assemblages in cluster D consisted of 50% individuals belonging to the Zodariidae family while exhibiting the lowest values for total abundance, richness and diversity (Figure 5). This family has been shown to be typical of open areas with low vegetation cover (Pekár et al. 2021), such as the sites in cluster D, which also displayed the highest proportion of surrounding sealed soils. Furthermore, Zodariidae species are exclusively ground‐dwelling spiders (Langlands et al. 2011). This strict epigeic lifestyle, combined with their lack of aerial dispersal capacity, severely limits their cursorial vagility. In these anthropogenically constrained yet temporally stable environments, such traits align with the characteristics of A‐strategists, which are selected to tolerate chronic environmental adversity. In addition, this cluster was also characterized by a very low abundance of the Lycosidae family (Figure 5), which may be linked to a very low herbaceous cover and a sandier soil texture (Figures 5 and 6). As demonstrated by Halloran et al. (2000), sandy soils can exclude Lycosidae due to soil instability. In contrast, high herbaceous cover and clay soil texture were the key features of the subsites in cluster B, where the majority of indicator species belonging to the Lycosidae family were found (Table 2). Other families exhibited variations in their distribution among clusters. For example, Linyphiidae were significantly more abundant in the clusters with little or no contamination (A–C) than in other clusters (D–F; Figure 5). This family experienced a decrease in abundance with increasing MI_Fe_Co_Ni_Cu values (Figure S13). In another context of metal contamination (Cd and Pb), Jung et al. (2008) highlighted a shift in Linyphiidae assemblages, supporting their sensitivity to these contaminants. These small spiders are considered to be r‐strategists due to their high aerial dispersal capacity via ballooning. Although this family was predominantly found in forested sites in our study (cluster A), Öberg et al. (2007) have shown that open agricultural habitats also promote their abundance and diversity. These observations reflect a trade‐off in Linyphiidae ecological preferences: while they require structurally complex microhabitats like forest litter and low vegetation for web construction, they rely on open spaces to launch into the air for ballooning (Gallé et al. 2020). Thus, although we highlighted a significant impact of certain metals on Linyphiidae abundance, vegetation cover and landscape structure remain the primary drivers of their distribution. Similar trends are documented for other faunal groups of soil organisms such as nematodes (Shao et al. 2012) or myriapods (Tudose and Rîșnoveanu 2024), for which vegetation cover and soil characteristics (other than contamination) are the main drivers of species distribution, as well as the taxonomic structure and composition of assemblages. Consequently, the results of our study have demonstrated that, while certain changes in the taxonomic composition of spider communities related to soil metal contamination, local environmental conditions and surrounding land‐uses exert a stronger influence. Finally, our findings contrast a selection of r‐strategists in the least contaminated broadleaved forested areas (e.g., the Linyphiidae family in cluster A subsites), with a dominance of A‐strategists in sealed and metal‐contaminated soils (e.g., the Zodariidae species in subsites of cluster D).

### 4.2 Environmental Parameters Such as Vegetation and Soil Physicochemistry Shape the Functional Traits of Spider Communities More Than Metal Contamination

We hypothesized a change in functional composition (hypothesis 3) and functional homogenization (hypothesis 4) of spider communities depending on environmental parameters and metal contamination. First, reference cluster A was predominantly represented by sheet‐web building spiders that feed on flying prey (lepidopterans, dipterans; Figure 8). These non‐sticky webs, which are often found in the low vegetation in forested areas, act as interception traps for flying or jumping prey. This method of capturing and consuming non‐specific prey can be considered a characteristic of r‐strategists used by generalist spiders, explaining the selection of euryphagous spiders in this cluster (Figure 8). This also constitutes a passive hunting strategy (Mannu et al. 2020; Roberts 2020; Schirmel et al. 2016), as well as the orb web spinning which was an indicator modality in the reference grasslands (cluster B). Furthermore, trophic transfer remains the primary route of metal exposure for spiders. For example, Perrotta et al. (2025) demonstrated that riparian spiders (Araneidae and Tetragnathidae) consuming insects emerging from the copper‐contaminated Torch Lake (Michigan, USA) exhibited significant copper bioaccumulation alongside an upregulation of lipid and xenobiotic metabolism driven by their microbiome. However, such disturbances also directly impair spider fitness. As shown for the orb‐web weaver *Araneus ventricosus* (Araneidae), consuming prey enriched in Cd (up to 15.78 μg/g) and Pb (up to 3129.49 μg/g) downregulates genes involved in the silk production pathway, leading to weakened webs or complete spinning failure (Yang et al. 2023). Under these laboratory conditions (in boxes with wooden frames), behavioral alterations have also been observed with *A. ventricosus* building webs closer to the ground. This shift could stem from a neurotoxic effect and altered cognitive or locomotor functions, similar to the loss of “episodic‐like memory” in the funnel‐web *Agelena labyrinthica* (Agelenidae) exposed to Hg (10 mg/L of Hg/(NO_3_)_2_ in drinking water; Liu et al. 2013). While data on these behavioral shifts remain scarce, such impairments likely reduce prey capture efficiency and negatively affect spider survival in the field. Conversely, the reference grasslands in cluster B were also characterized by a high abundance of cursorial hunters, which actively pursue their prey on the ground. This ability enables them to disperse more easily through the vegetation (Öberg et al. 2007). Moreover, the herbaceous cover could provide favorable conditions for the development of some of their prey, such as orthopterans (a key prey type for this cluster; Schirmel et al. 2011), and could allow the spiders to detect them more easily thanks to the vibrations caused by the prey moving through the vegetation (Japyassú and Laland 2017). Conversely, we observed a significant decrease in functional evenness only within cluster B (Figure 7), suggesting that the spider communities in this cluster exhibit a more heterogeneous use of trait modalities. This could be due to the dominance (79% of the individuals in subsites of this cluster) of the Lycosidae family, whose species share relatively similar trait combinations (e.g., cursorial spiders, maternal care).

On the other hand, functional richness (Villéger et al. 2008) depends on the diversity of trait combinations present simultaneously within spider communities. It therefore appeared that, within subsites of cluster D, the low values observed for taxonomic richness and taxonomic diversity were also accompanied by a low functional richness and a high homogeneity in the trait modalities used by spiders (Figure 7). The sites of this cluster exhibited the highest proportion of sealed land‐cover within a 50‐m radius around the subsites (Figure 3). These environmental filters selected for stenophagous spiders that build retreats under stones and actively feed on Hymenoptera. Most of the Hymenoptera were ants. Ants are indeed more abundant and diverse in open areas with sparse vegetation than in forested areas, and soil sealing can actively promote the selection of specialized ant species that serve as reliable food source of spiders (Holec and Frouz 2005; Melliger et al. 2018). This specialized diet, coupled with a specific microhabitat requirement in a temporally stable yet highly contaminated environment further supports the selection of A‐strategists. Moreover, these functional trait combinations were positively associated with high C_org_/N ratio values (Figure 7). Zhou et al. (2016) demonstrated that metal‐contaminated environments exhibited (i) a decrease in the amount of organic carbon and nitrogen, but (ii) an increase in the C_org_/N ratio, as observed in the subsites of cluster D. This ratio is an important explanatory parameter because nitrogen is one of the main components of the amino acids that compose spider silk (Ott et al. 2014). Sanders et al. (2015) found that ground‐living spiders are more enriched in carbon than orb web weavers, which are richer in nitrogen, because they build their webs higher in vegetation and capture insects richer in nitrogen. This tends to validate the indicator value of the ground hunter modality for cluster D and the indicator value of the orb web modality for cluster B. Similarly, cluster E was represented by spiders that do not build web but rather retreat in vegetation and/or under bark. These modalities appeared to be strongly related to high pH values. Indeed, it has been shown that a slightly acidic pH is a determining factor in the strength and elasticity of web proteins and that these proteins are extremely soluble at neutral to basic pH values (Andersson et al. 2016). Neutral to basic conditions, such as those found in the subsites of cluster E, could weaken webs, rending them ineffective as permanent shelters or hunting tools. However, these conditions might be sufficient to allow spiders to build small temporary shelters such as retreats. This theory is also supported by the fact that, conversely, web construction (e.g., sheet‐web) was associated with acidic pH levels (Figure 8). Although, to our knowledge, no field study has clearly established a link between soil pH and spider web structure, these findings open up interesting avenues for future research.

Therefore, mirroring the patterns observed with the taxonomic approach, while soil metal contamination contributes to shaping the functional structure and trait composition of spider communities, other soil characteristics such as vegetation cover, soil granulometry, pH and the C_org_/N ratio exert a stronger influence on the observed trait variations. Land‐cover surrounding the subsites also plays a decisive role in the selection of r‐strategists in the reference forests (cluster A) and grasslands (cluster B), whereas heavily sealed areas (e.g., in cluster D) predominantly select for A‐strategists.

### 4.3 Taxonomic Approach or Trait‐Based Approach: Which Is the Best Strategy for Studying Spider Communities in Contaminated Environments?

Interestingly, the proportion of variation in spider assemblages explained by environmental variables was higher for the trait‐based analysis than for the taxon‐based analysis. Our results therefore confirm the relevance of the trait‐based approach for studying the response of spider communities to anthropogenic pressures, but this approach requires detailed knowledge of the traits, which is not always feasible as it can be costly and requires specialized expertise (Hodkinson and Jackson 2005). The use of “potential traits” described from information in the literature, as in our study, is an interesting alternative to the effort and time required for direct measurement (i.e., “realized traits”). Indeed, while approaches based on realized traits provide access the actual responses of organisms to environmental pressures, they can only be deployed on a “small scale” because these methods are very time‐consuming and difficult to apply in practice to a large number of traits, species, and sites. Using traits resulting from a literature review of known biological information on species in a wide range of environmental conditions (= potential traits) then becomes an effective alternative for identifying the main trends in the combinations of adaptations selected within communities under anthropogenic constraints, when working with diverse communities (219 taxa in our study), sampled across a large number of sites (40 sites in our study), and considering several traits (7 in our study, but 32 traits described by 77 modalities in the initial database). In terms of bioindication and bioremediation, the simultaneous observation of biological traits and environmental descriptors make it easier to identify the traits/habitat properties ensuring the resistance and resilience of communities and ecosystems (Walker 1992). As already highlighted in Hodkinson and Jackson (2005) and Ross et al. (2022), this approach allows to identify trait syndromes—i.e., combinations of trait modalities selected simultaneously in spider assemblages—in metal‐contaminated environments. In particular, we have highlighted the importance of tracking the type of web constructed, the hunting guild, and the type of prey hunted. However, while species pools naturally vary on a large spatial scale (e.g., at the level of land patches, regions or continent), given the respective ranges of their species, functions—and therefore traits—remain, which makes trait‐based approaches much less dependent of the biogeographical context than taxonomic approaches (Vandewalle et al. 2010). By analyzing the structure and composition of spider communities in 40 wastelands located along a metal contamination gradient, we have highlighted the importance of combining taxonomic and functional data in this analysis, as they provide complementary information. From this perspective, it would be interesting to spatially extend the present study (despite a geographical scale already covering more than 300 km) to examine the degree to which responses to metal contamination are similar in other environmental contexts.

### 4.4 Considering the Real Exposure of Spider Communities: Metal Bioavailability and Resistance Mechanisms in Spiders

The physicochemical properties or long‐term aging contamination of the soils studied could have resulted in low metal bioavailability of metals and therefore, a limited effect of metal concentrations on the taxonomic composition and functional profiles of spider communities. The pH is the main determinant of metal bioavailability and mobility (Basta et al. 2005). Indeed, low pH values tend to promote the speciation of metals in their ionic form, the fraction considered bioavailable due to its ability to bind to an organic ligand (Basta et al. 2005). Conversely, neutral to basic pH values, such as those measured in the soils of subsites in clusters B–F, tend to keep metals in inorganic form (Asare and Afriyie 2021; Basta et al. 2005), making them less likely to impact soil macroinvertebrates through contact or trophic uptake. Moreover, the taxonomic structure of spider assemblages was significantly influenced by CaCO_3_, with the highest concentrations measured in clusters D and F (Figure 2 and Figure S5). A previous study conducted in Pompey (site no. 15 in cluster F, see Table S1) showed that calcite is a product of sludge discharge into settling basins and that it forms as this material evolves under the influence of environmental parameters (Huot et al. 2013). This calcite, which ensures spiders' cuticle hardness, may be less available in acidic environments (Glime 2017). It would be interesting to determine the specific requirements (in particular for calcite) of spider species captured in the most acidic soils (e.g., sites of the cluster A) for the production of their cuticle, and to compare these requirements with those of species captured in soils with a neutral or alkaline pH. CaCO_3_ is also known to be an important buffering agent for metals, and thus a key factor in their bioavailability in soils (e.g., Gee et al. 2001; Wang et al. 2015).

The age of the contamination is also known to be a factor limiting metal mobility (Ma et al. 2006). Over time, soluble elements are leached out, leaving only elements that are attached to soil particles (Smolders et al. 2009). In particular, metals can bind on clay particles, immobilizing them in the soil (Madrid et al. 2008). We also highlighted an important effect of vegetation and surrounding land‐cover on taxonomic and functional structure of spider assemblages. This trend is further supported by the correlation between vegetation variables and synthetic landscape descriptors, especially for the “forest to grassland” gradient (Figure 2). After a mine is abandoned, an ecological succession of vegetation occurs. Spiders can act as pioneer species and recolonize these contaminated environments, even when they are still devoid of vegetation or have only sparse vegetation, as observed in sites of cluster D (Mrzljak and Wiegleb 2000; Walter et al. 2024). Subsequently, as the vegetation cover develops and diversifies, the overall richness of invertebrate species increases, potentially implying a greater and more diverse availability of prey for spiders (Hendrychová et al. 2012). Furthermore, studies conducted by Skalski et al. (2010) and Żmudzki and Laskowski (2012) have shown that there is not necessarily competition between certain predators (namely spiders and beetles) in contaminated environments. It would be interesting to verify this at our sites, particularly with regard to interactions between spiders and predatory beetles of the Carabidae family. However, it is possible that the vegetation structure at metal‐contaminated sites ensures a sufficient supply of prey and thus little or no competition among predators, which could also mitigate the effects of metal contamination. Later in ecological succession, trees can colonize areas contaminated by metals (e.g., Huot et al. 2013). Along transects linking grasslands to forests, Grim et al. (2021) demonstrated that web‐weaver spiders were found exclusively in forests. Variations in vegetation cover can therefore induce changes in functional traits within communities. Thus, both grassland covers and forest cover are important predictors of the taxonomic and functional diversity of spiders communities. However, anthropized areas must also be taken into account. Magura et al. (2010) found greater spider diversity in sealed and urbanized areas due to the presence of grasslands and arable lands in the surrounding landscape. In contrast, we observed no significant variation in taxonomic metrics at subsites with sealed soils (from clusters C, E and F), despite the diversity of the surrounding landscape within a 500‐m radius. However, a negative relationship between the presence of sealed soil at the subsite scale and the abundance of web‐building spiders was found, which is consistent with Magura et al. (2010) and suggests that these spiders of forested areas are sensitive to soil sealing. Next, four of the studied metals showed concentration above the Eco‐SSL threshold: in addition to Zn and Pb already mentioned, 30 subsites exceeded 80 μg/g of Cu (range of values from 1.2 to 284.5 μg/g) and 104 exceeded 280 μg/g of Mn (from 29.9 to 11,127.4 μg/g; US EPA 2015). Such concentration ranges can have a negative impact on invertebrates. For example, ingesting soil containing 176 μg/g of Cu leads to a significant decrease in earthworm abundances (Creamer et al. 2008; Nahmani and Lavelle 2002), which means that spiders are potentially less exposed and/or more resistant to metal contamination than certain other invertebrates. This combination of physicochemical and landscape factors could explain the absence of a metal index having a major structuring effect on spider communities, despite the high total concentrations of metals in the soils of in this study (see Table S1). On the other hand, spiders can be exposed to metals directly through soil solution and indirectly through trophic intake (Żmudzki and Laskowski 2012). Soliman et al. (2022) highlighted how *Hogna ferox* (Lycosidae), relative to its biomass, bioaccumulated a higher proportion of metals from the consumption of contaminated prey than other species of the studied food web across a wide variety of industrial contexts (e.g., petroleum coke, cement factories, metallurgy). Several studies carried out on various species have confirmed the spiders' ability to bioaccumulate, particularly through dietary exposure (Aziz et al. 2020; Hansson et al. 2019; Hendrickx et al. 2004), while others have highlighted complementary mechanisms such as metal excretion (Hopkin 1989) or detoxification capacity (Babczyńska et al. 2006; Wilczek et al. 1997), which enable spiders to survive in metal‐contaminated environments. Spiders are also mobile organisms with a high dispersal ability which allows them to potentially limit their direct and indirect exposure. This could explain the limited effects of metal concentrations observed in this study. Unfortunately, the dispersal abilities of spiders are not yet readily available in current databases on spider traits. Based on fuzzy‐coded trait information from the literature, it is difficult for now to determine the actual importance of a given dispersal ability strategy (e.g., the ballooning capacity) in limiting the effects of contamination on the species that use it. This is all the more true given that some species can use it at all stages of their development, while others do so only during the juvenile stage (Mrzljak and Wiegleb 2000), information that was not recorded during trait coding. Given the differences observed in spiders' dispersal abilities depending on their developmental stage, it would be interesting to assess more precisely the selective impact of metal contamination on their dispersal strategies as a part of a landscape scale study.

## 5 Conclusion

Approaches based on both taxonomic and trait‐based characteristics of assemblages are still under development for terrestrial invertebrates, although they have evolved considerably over the past 15 years. Here, we present a comprehensive application of these combined approaches to spiders, a ubiquitous, diverse, and abundant group of soil invertebrates. By analyzing the structure and composition of spider communities in 40 wastelands along a metal contamination gradient, we have highlighted the importance of combining taxonomic and functional information in this analysis. We have demonstrated that metal contamination contributes, without being the main driver, to shaping the taxonomic and functional structures of spider communities. Other environmental parameters such as vegetation cover, soil granulometry, pH, CaCO_3_ or C_org_/N ratio were also highly structuring. It is also important to consider the landscape structure within a 50‐ and 500‐m radius around the sites, particularly the extent of broadleaved forest cover, permanent herbaceous cover, and sealed surface cover. We identified relevant indicator species and families for groups of sites along the metal contamination gradient, including 14 species of uncontaminated environments versus only three in more contaminated ones. *X. nemoralis* (Lycosidae) is an example: this species is able to maximize egg survival in stable environments heavily contaminated with metals, making it a typical A‐strategist. On the other hand, we identified trait syndromes combining euryphagy—with a wide variety of different prey consumed—and web‐building at reference subsites (i.e., characteristics typically associated with r‐strategists). Conversely, ground‐hunting, non‐web‐building, as well asmore specialized diet (particularly an ant‐based diet considered to be a trait characteristic of A‐strategists) were subject to stronger selection within spider communities in the most contaminated areas. Finally, functional traits better explained the observed variations in our dataset than taxonomic composition. This result supports the application of a trait‐based approach in future studies on spiders, which will benefit from our new trait database. In this regard, it would seem particularly relevant to focus on the traits “web type”, “hunting strategy”, and “prey type”. A better understanding of the impact of natural environmental characteristics and anthropogenic pressures on soil communities would allow us to better understand the functioning of the soil ecosystems and would facilitate the development of more effective strategies to protect or restore them.

## Author Contributions

**Aliénor Duval:** formal analysis (lead), investigation (equal), writing – original draft (lead). **Vincent Laderriere:** formal analysis (lead), investigation (equal), writing – review and editing (equal). **Margot Jans:** investigation (equal), writing – review and editing (equal). **Jonathan Bouquerel:** investigation (equal). **Philippe Usseglio‐Polatera:** conceptualization (lead), investigation (equal), methodology (lead), writing – review and editing (equal). **Aurélie Cebron:** conceptualization (lead), investigation (equal), methodology (lead), writing – review and editing (equal). **Florence Maunoury‐Danger:** conceptualization (lead), funding acquisition (lead), investigation (equal), methodology (lead), writing – review and editing (equal).

## Funding

This work was supported by the French Ministry of Education. Agence de l'Environnement et de la Maîtrise de l'Energie (ADEME), DiagnoTraits program; 2172D0218‐A. EC2CO CNRS/INSU program, AT Microbiome; project DiagnoBactO. Zone Atelier Moselle (ZAM).

## Conflicts of Interest

The authors declare no conflicts of interest.

## Supporting information


**Figure S1:** Organization of the sampling sites and subsites, with the locations of pitfall traps used to collect spiders, samples for soil analyses, measurements of herbaceous cover such as plant biomass and the location from which the hemispherical picture of each subsite was taken.
**Figure S2:** Land‐cover within a five‐meter radius around each subsite. It was described using the Corine Land Cover Backbone, version 2018, which has a resolution of 10 m at the European scale. Within this five‐meter radius, the areas of the different land‐cover categories were determined by pixel counting and then converted into percentage coverage. In this representation, the subsites were grouped into clusters (A–F), as described previously.
**Figure S3:** Land‐cover within a 50‐m radius around each subsite. It was described using the Corine Land Cover Backbone, version 2018, which has a resolution of 10 m at the European scale. Within this 50‐m radius, the areas of the different land‐cover categories were determined by pixel counting and then converted into percentage coverage. In this representation, the subsites were grouped into clusters (A–F), as described previously.
**Figure S4:** Land‐cover within a 500‐m radius around each subsite. It was described using the Corine Land Cover Backbone, version 2018, which has a resolution of 10 m at the European scale. Within a 500‐m radius, the areas of the different land‐cover categories were determined by pixel counting and then converted into percentage coverage. In this representation, the subsites were grouped into clusters (A–F) as described previously.
**Figure S5:** Comparison of environmental parameters among the six clusters of sites. Clusters correspond to subsites belonging to the same group according to a classification based on environmental parameters (see the “clustering of sites” subsection for more details). Significant differences were assessed using Kruskal–Wallis tests, followed by Dunn *post hoc* tests with Bonferroni *p*‐value adjustment. Different letters indicate clusters with significant differences for a given index (*p*‐value < 0.05).
**Figure S6:** Contribution of each environmental parameter (%) to the definition of the first three principal components of the PCA applied to the table of 120 subsites described by 17 environmental parameters (see the results of the PCA in Figure 2).
**Figure S7:** Results of the PCA analysis of the 120 subsites, based on a description of the land‐cover within a radius of 5, 50 and 500 m around them. (A) Distribution of subsites belonging to the six clusters (A–F) and (B) correlations between environmental parameters and subsite coordinates along the first two principal components. The eight land‐cover categories identified around the 120 subsites were used: sealed soils, woody needle or broadleaved deciduous trees, low‐growing woody plants (e.g., bushes), permanent or periodically herbaceous, non and sparsely vegetated and water. The percentage cover of each land‐cover category was calculated at the three observation scales (except for “water”, which was not detected on any subsite within a 5‐m radius). On panel B, each color corresponds to one of the land‐cover categories.
**Figure S8:** Contribution of each land‐cover category (%) within a 5‐, 50‐ and 500‐m radius around the 120 subsites, to the definition of the first two principal components of the PCA applied to the table of 120 subsites described by the percentage coverage of 23 parameters based on land‐cover (cf. Figure S7). The eigenvalue of each axis is indicated in the title of the graph.
**Figure S9:** Generalized Additive Model (GAM) highlighting the significant impact of eight environmental parameters on the total abundance of spiders at the subsites. Given the wide variation in values of MIs and PAHs, these parameters were log10‐transformed. Next, the parameters were automatically selected to minimize the AIC (i.e., to ensure that the model is as efficient as possible). Five environmental parameters exhibited a nonlinear relationship with total abundance (panels A–E) and were subsequently smoothed by the model. For the corresponding parameters, the numerical value shown on the y‐axis (in parentheses) corresponds to the estimated number of degrees of freedom. Three parameters presented a linear relationship with total abundance (panels F–H). Fifty‐six percent of the variance was explained by these eight parameters.
**Figure S10:** Generalized Additive Model (GAM) highlighting the significant impact of ten environmental parameters on the taxonomic richness of spider assemblages at the subsites. Given the wide variation in values of MIs and PAHs, these parameters were log10‐transformed. Next, the parameters were automatically selected to minimize the AIC (i.e., to ensure that the model is as efficient as possible). Seven environmental parameters exhibited a nonlinear relationship with taxonomic richness (A–G) and were subsequently smoothed by the model. For the corresponding parameters, the numerical value shown on the y axis (in parentheses) corresponds to the estimated number of degrees of freedom. Three parameters presented a linear relationship with taxonomic richness (H–J). Eighty percent of the variance was explained by these ten parameters.
**Figure S11:** Generalized Additive Model (GAM) highlighting the significant impact of seven environmental parameters on the taxonomic diversity of spider assemblages at the subsites. Given the wide variation in values of MIs and PAHs, these parameters were log10‐transformed. Next, the parameters were automatically selected to minimize the AIC (i.e., to ensure that the model is as efficient as possible). Four environmental parameters exhibited a nonlinear relationship with taxonomic diversity (A–D) and were subsequently smoothed by the model. For the corresponding parameters, the numerical value shown on the y axis (in parentheses) corresponds to the estimated number of degrees of freedom. Three parameters presented a linear relationship with taxonomic diversity (E–G). Fifty‐five percent of the variance was explained by these seven parameters.
**Figure S12:** Generalized Additive Model (GAM) highlighting the significant impact of five environmental parameters on the abundance of Lycosidae at the subsites. Given the wide variation in values of MIs and PAHs, these parameters were log10‐transformed. Next, the parameters were automatically selected to minimize the AIC (i.e., to ensure that the model is as efficient as possible). Three environmental parameters exhibited a nonlinear relationship with Lycosidae abundance (A–C) and were subsequently smoothed by the model. For the corresponding parameters, the numerical value shown on the y axis (in parentheses) corresponds to the estimated number of degrees of freedom. Two parameters presented a linear relationship with Lycosidae abundance (D, E). Fifty‐four percent of the variance was explained by these five parameters.
**Figure S13:** Generalized Additive Model (GAM) highlighting the significant impact of four environmental parameters on the abundance of Linyphiidae at the subsites. Given the wide variation in values of MIs and PAHs, these parameters were log10‐transformed. Next, the parameters were automatically selected to minimize the AIC (i.e., to ensure that the model is as efficient as possible). All the four environmental parameters exhibited a nonlinear relationship with Linyphiidae abundance and were subsequently smoothed by the model. For the corresponding parameters, the numerical value shown on the y axis (in parentheses) corresponds to the estimated number of degrees of freedom. Forty‐five percent of the variance was explained by these four parameters.

**Table S1:** Physico‐chemical characterization of the 40 sites sampled during the DiagnoTraits project. Each site was divided into three subsites. The values shown for each site correspond to the minimum and maximum values measured across all three subsites.

**Table S2:** Fuzzy‐coding trait profiles for seven functional traits of 194 taxa of spiders. Cells are filled with 0 (no affinity) to 3 (maximum affinity). The empty cells correspond to the uncoded traits, due to a lack of information in the existing literature. See Table 1 for the full labels of the trait modality codes and to see which modalities were grouped for the analyses.

**Data S1:** Bibliographic references for the sources of information used to compile the database on the functional traits of spiders.

## Data Availability

Data and scripts are available at the following link: https://doi.org/10.5281/zenodo.20624199.
