# Supplementary material for: Functional and Taxonomic Structuring of Ground‐Dwelling Spider Communities Under the Combined Effects of the Environment and Metal Contamination
Source: Ecol Evol. 2026 Aug 18;16(8):e73958. doi: 10.1002/ece3.73958 (PMC13487381; doi:10.1002/ece3.73958)
Supplement: Supplementary file 1 [file ECE3-16-e73958-s001.docx]

**Figure S1:** **Organization of the sampling sites and subsites**, with the locations of pitfall traps used to collect spiders, samples for soil analyses, measurements of herbaceous cover such as plant biomass and the location from which the hemispherical picture of each subsite was taken.

**Figure S2: Land-cover within a five-meter radius around each subsite.** It was described using the Corine Land Cover Backbone, version 2018, which has a resolution of 10 meters at the European scale. Within this five-meter radius, the areas of the different land-cover categories were determined by pixel counting and then converted into percentage coverage. In this representation, the subsites were grouped into clusters (A to F), as described previously.

**Figure S3: Land-cover within a fifty-meter radius around each subsite.** It was described using the Corine Land Cover Backbone, version 2018, which has a resolution of 10 meters at the European scale. Within this fifty-meter radius, the areas of the different land-cover categories were determined by pixel counting and then converted into percentage coverage. In this representation, the subsites were grouped into clusters (A to F), as described previously.

**Figure S4: Land-cover within a five-hundred-meter radius around each subsite.** It was described using the Corine Land Cover Backbone, version 2018, which has a resolution of 10 meters at the European scale. Within a five-hundred-meter radius, the areas of the different land-cover categories were determined by pixel counting and then converted into percentage coverage. In this representation, the subsites were grouped into clusters (A to F) as described previously.

**Figure S5: Comparison of environmental parameters among the six clusters of sites.** Clusters correspond to subsites belonging to the same group according to a classification based on environmental parameters (see the “clustering of sites” subsection for more details). Significant differences were assessed using Kruskal-Wallis tests, followed by Dunn *post-hoc* tests with Bonferroni p-value adjustment. Different letters indicate clusters with significant differences for a given index (p-value < 0.05).

**Figure S6:** Contribution of each environmental parameter (%) to the definition of the first three principal components of the PCA applied to the table of 120 subsites described by 17 environmental parameters (see the results of the PCA in Figure 2).

**Figure S7:** **Results of the PCA analysis of the 120 subsites, based on a description of the land-cover within a radius of 5, 50 and 500m around them.** A) Distribution of subsites belonging to the six clusters (A to F) and B) correlations between environmental parameters and subsite coordinates along the first two principal components. The eight land-cover categories identified around the 120 subsites were used: sealed soils, woody needle or broadleaved deciduous trees, low-growing woody plants (e.g., bushes), permanent or periodically herbaceous, non and sparsely vegetated and water. The percentage cover of each land-cover category was calculated at the three observation scales (except for “water”, which was not detected on any subsite within a 5-meter radius). On panel B, each color corresponds to one of the land-cover categories.

**Figure S8**: Contribution of each land-cover category (%) within a 5-, 50- and 500-meter radius around the 120 subsites, to the definition of the first two principal components of the PCA applied to the table of 120 subsites described by the percentage coverage of 23 parameters based on land-cover (cf. Fig. S7). The eigenvalue of each axis is indicated in the title of the graph.

**Figure S9:** Generalized Additive Model (GAM) highlighting the significant impact of eight environmental parameters on the total abundance of spiders at the subsites. Given the wide variation in values of MIs and PAHs, these parameters were log10-transformed. Next, the parameters were automatically selected to minimize the AIC (i.e., to ensure that the model is as efficient as possible). Five environmental parameters exhibited a nonlinear relationship with total abundance (panels A to E) and were subsequently smoothed by the model. For the corresponding parameters, the numerical value shown on the y-axis (in parentheses) corresponds to the estimated number of degrees of freedom. Three parameters presented a linear relationship with total abundance (panels F to H). Fifty-six percent of the variance was explained by these eight parameters.

**Figure S10:** Generalized Additive Model (GAM) highlighting the significant impact of ten environmental parameters on the taxonomic richness of spider assemblages at the subsites. Given the wide variation in values of MIs and PAHs, these parameters were log10-transformed. Next, the parameters were automatically selected to minimize the AIC (i.e., to ensure that the model is as efficient as possible). Seven environmental parameters exhibited a nonlinear relationship with taxonomic richness (panels A to G) and were subsequently smoothed by the model. For the corresponding parameters, the numerical value shown on the y axis (in parentheses) corresponds to the estimated number of degrees of freedom. Three parameters presented a linear relationship with taxonomic richness (panels H to J). Eighty percent of the variance was explained by these ten parameters.

**Figure S11:** Generalized Additive Model (GAM) highlighting the significant impact of seven environmental parameters on the taxonomic diversity of spider assemblages at the subsites. Given the wide variation in values of MIs and PAHs, these parameters were log10-transformed. Next, the parameters were automatically selected to minimize the AIC (i.e., to ensure that the model is as efficient as possible). Four environmental parameters exhibited a nonlinear relationship with taxonomic diversity (panels A to D) and were subsequently smoothed by the model. For the corresponding parameters, the numerical value shown on the y axis (in parentheses) corresponds to the estimated number of degrees of freedom. Three parameters presented a linear relationship with taxonomic diversity (panels E to G). Fifty-five percent of the variance was explained by these seven parameters.

**Figure S12:** Generalized Additive Model (GAM) highlighting the significant impact of five environmental parameters on the abundance of Lycosidae at the subsites. Given the wide variation in values of MIs and PAHs, these parameters were log10-transformed. Next, the parameters were automatically selected to minimize the AIC (i.e., to ensure that the model is as efficient as possible). Three environmental parameters exhibited a nonlinear relationship with Lycosidae abundance (panels A to C) and were subsequently smoothed by the model. For the corresponding parameters, the numerical value shown on the y axis (in parentheses) corresponds to the estimated number of degrees of freedom. Two parameters presented a linear relationship with Lycosidae abundance (panels D and E). Fifty-four percent of the variance was explained by these five parameters.

**Figure S13:** Generalized Additive Model (GAM) highlighting the significant impact of four environmental parameters on the abundance of Linyphiidae at the subsites. Given the wide variation in values of MIs and PAHs, these parameters were log10-transformed. Next, the parameters were automatically selected to minimize the AIC (i.e., to ensure that the model is as efficient as possible). All the four environmental parameters exhibited a nonlinear relationship with Linyphiidae abundance and were subsequently smoothed by the model. For the corresponding parameters, the numerical value shown on the y axis (in parentheses) corresponds to the estimated number of degrees of freedom. Forty-five percent of the variance was explained by these four parameters.
