# Supplementary material for: Functional and Taxonomic Structuring of Ground‐Dwelling Spider Communities Under the Combined Effects of the Environment and Metal Contamination
Source: Ecol Evol. 2026 Aug 18;16(8):e73958. doi: 10.1002/ece3.73958 (PMC13487381; doi:10.1002/ece3.73958)
Supplement: Supplementary file 2 [file ECE3-16-e73958-s004.docx]

**Bibliographic references for the sources of information used to compile the database on the functional traits of spiders:**

1. **BOOKS**

Becker, L. (1882). Arachnides de Belgique (Vol. 10). Académie Royale de Belgique, Brussels, Belgium.

Corfdir, V. (2022). Guide pratique des insectes, 3^rd^ edition, France Agricole.

Harvey, P. R., Nellist, D. R., & Telfer, M. G. (2002). Provisional Atlas of British Spiders (Arachnida, Araneae) (New edition, Vol. 2). Biological Records Centre, Centre for Ecology & Hydrology, Huntingdon, UK.

Jocqué, R., & Dippenaar-Schoeman, A. S. (2007). Spider Families of the World, 2^nd^ edition, MRAC Musée royal de l’Afrique centrale.

Kammenga, J., & Laskowski, R. (2000). Demography in Ecotoxicology. Ecological & Environmental Toxicology Series, John Wiley & Sons, Chichester, England.

Martiré, D., Merlier, F., & Eyssartier, G. (2021). L’indispensable guide des araignées de France et autres arachnides (Illustrated edition). Belin, France.

Rainer, F. (2010). Biology of Spiders, 3^rd^ edition, Oxford University Press, USA.

Roberts, M. J. (2020). Araignées de France et d’Europe. Delachaux & Niestlé.

Rollard, C., Blanchot, P., & Villani, C. (2014). Portraits d’araignées. Quae, France

Sandhall, Å. (1975). Insectes et petites bêtes : Dans leur milieu naturel. Nathan, France.

Savory, T. H. (1928). The Biology of Spiders. Sidgwick & Jackson, London, UK.

Sentenac, G. (2011). La faune auxiliaire des vignobles de France. France Agricole.

2. **DATABASES**

Bach, A., Raub, F., Höfer, H., Ottermanns, R., & Roß-Nickoll, M. (2024). ARAapp: Filling gaps in the ecological knowledge of spiders using an automated and dynamic approach to analyze systematically collected community data. Database, 2024, baae004. <https://doi.org/10.1093/database/baae004>

GBIF.org (2024), GBIF Home Page. https://www.gbif.org

Joimel, S., Nahmani, J., Hedde, M., Auclerc, A., Beaumelle L., … & Pey, B. (2021). A large database on functional traits for soil ecologists: BETSI. Global Symposium on Soil Biodiversity, FAO, Apr 2021, Rome, Italy. pp.523-528.

MNHN & OFB [Ed]. 2003-2024. Inventaire national du patrimoine naturel (INPN), Site web: https://inpn.mnhn.fr

Nentwig, W., Blick, T., Bosmans, R., Gloor, D., Hänggi, A., & Kropf, C. (2010). Spiders of Europe. <https://doi.org/10.24436/1>

Pekár, S., Wolff, J. O., Černecká, Ľ., Birkhofer, K., Mammola, S., Lowe, E. C., Fukushima, C. S., Herberstein, M. E., Kučera, A., Buzatto, B. A., Djoudi, E. A., Domenech, M., Enciso, A. V., Piñanez Espejo, Y. M. G., Febles, S., García, L. F., Gonçalves-Souza, T., Isaia, M., Lafage, D., … & Cardoso, P. (2021). The World Spider Trait database: A centralized global open repository for curated data on spider traits. Database, 2021. https://doi.org/10.1093/database/baab064

University of Michigan, Museum of Zoology. (s. d.). Animal Diversity Web. https://animaldiversity.org/

Webb, J., Heaver, D., Lott, D., Dean, H. J., van Breda, J., Curson, J., Harvey, M. C., Gurney, M., Roy, D. B., van Breda, A., Drake, M., Alexander, K. N. A., & Foster, G. (2018). Pantheon. https://pantheon.brc.ac.uk/

3. **PAPERS**

Agnarsson, I. (2022). Elevation gradient affects the distribution and host utilisation of *Zatypota anomala* (Hymenoptera, Ichneumonidae) associated with mesh web weaving spiders (Araneae, Dictynidae). Journal of Hymenoptera Research, 93, 89‑100. <https://doi.org/10.3897/jhr.93.91513>

Aitchison, C. W. (1984). Low Temperature Feeding by Winter-Active Spiders. The Journal of Arachnology, 12(3), 297‑305.

Alzubik Belkair, S., Mazzia, C., Pasquet, A., & Capowiez, Y. (2018). Temporal activity of spiders and earwigs during winter in apple trees under a Mediterranean climate. Biocontrol Science and Technology, 28(9), 823‑836. <https://doi.org/10.1080/09583157.2018.1499870>

Assi Bessékon, D. (1997). Intraspecific identification and tolerance in the social-maternal behaviour of *Coelotes terrestris* (Araneae, Agelenidae). Behavioural Processes, 39(3), 231‑239. <https://doi.org/10.1016/S0376-6357(96)00756-5>

Babczyńska, A., Wilczek, G., & Migula, P. (2006). Effects of dimethoate on spiders from metal pollution gradient. Science of the Total Environment, 370(2‑3), 352‑359. <https://doi.org/10.1016/j.scitotenv.2006.06.024>

Bell, J. R., Bohan, D. A., Shaw, E. M., & Weyman, G. S. (2005). Ballooning dispersal using silk: World fauna, phylogenies, genetics and models. Bulletin of Entomological Research, 95(2), 69‑114. <https://doi.org/10.1079/BER2004350>

Birkhofer, K., Smith, H. G., Weisser, W. W., Wolters, V., & Gossner, M. M. (2015). Land‐use effects on the functional distinctness of arthropod communities. Ecography, 38, 889‑900. <https://doi.org/10.1111/ecog.01141>

Buchli, H. (1965). La conquête de terriers de mygales maçonnes par *Haplodrassus signifer* (Koch) Araignée Gnaphoside. Vie et Milieu, 593‑598.

Cabon, V., Pincebourde, S., Colinet, H., Dubreuil, V., Georges, R., Launoy, M., Pétillon, J., Quénol, H., & Bergerot, B. (2023). Preferred temperature in the warmth of cities: Body size, sex and development stage matter more than urban climate in a ground-dwelling spider. Journal of Thermal Biology, 117, 103706. <https://doi.org/10.1016/j.jtherbio.2023.103706>

Chatzaki, M. (2021). Description of New Ground Spider Species (Gnaphosidae, Araneae) from Mainland Greece. Taxonomy, 1(4), 374‑394. <https://doi.org/10.3390/taxonomy1040028>

Cooke, J. A. L. (1965). A Contribution to the Biology of the British Spiders belonging to the Genus *Dysdera*. Oikos, 16(1/2), 20-25. <https://doi.org/10.2307/3564861>

Déjean, S. (2020). *Agyneta inermis* Tanasevitch, 2019 (Araneae, Linyphiidae) une nouvelle espèce méditerranéenne pour la faune de France, avec description de la femelle. Revue arachnologique, 2(7), 23-26.

Delfosse, E. (1999). Notes sur la biologie et l’élevage de la petite Mygale française *Atypus affinis* Eichwald, 1830 (Arachnida Mygalomorphae Atypidae). Le bulletin de l’Association Phyllie, 1, 25-27.

Delfosse, E. (2005). Une chaussette à double embranchement chez une *Atypus affinis* Eichwald, 1830 (Arachnida, Araneae, Mygalomorphae, Atypidae). Le bulletin d’Arthropoda, 23, 50-51.

Dolejš, P., Kubcová, L., & Buchar, J. (2008). Subterrestrial life of *Arctosa lutetiana* (Araneae, Lycosidae). Journal of Arachnology, 36(1), 202‑203. <https://doi.org/10.1636/St07-33SC.1>

Dondale, C. D. (1961). Life histories of some common spiders from trees and shrubs in Nova Scotia. Canadian Journal of Zoology, 39(6), 777‑787. <https://doi.org/10.1139/z61-074>

Edgar, W. D. (1969). Prey and predators of the Wolf spider *Lycosa lugubris*. Journal of Zoology, 159(4), 405‑411. <https://doi.org/10.1111/j.1469-7998.1969.tb03897.x>

Edgar, W. D. (1972). The life‐cycle of the Wolf spider *Pardosa lugubris* in Holland. Journal of Zoology, 168(1), 1‑7. <https://doi.org/10.1111/j.1469-7998.1972.tb01336.x>

Foxová, D. (2023). Maternal care in spiders. Bachelor’s thesis, Charles University, Prague, CZ.

Gallé, R., Császár, P., Makra, T., Gallé-Szpisjak, N., Ladányi, Z., Torma, A., Ingle, K., & Szilassi, P. (2018). Small-scale agricultural landscapes promote spider and ground beetle densities by offering suitable overwintering sites. Landscape Ecology, 33(8), 1435‑1446. <https://doi.org/10.1007/s10980-018-0677-1>

Graca, B., Jędruch, A., Bełdowska, M., Bełdowski, J., Kotwicki, L., Siedlewicz, G., Korejwo, E., Popińska, W., & Łukawska-Matuszewska, K. (2022). Effects of beach wrack on the fate of mercury at the land-sea interface – A preliminary study. Environmental Pollution, 315, 120394. <https://doi.org/10.1016/j.envpol.2022.120394>

Gundermann, J., Horel, A., & Roland, C. (1997). Costs and Benefits of Maternal Care in a Subsocial Spider, *Coelotes terrestris*. Ethology, 103(11), 915‑925. <https://doi.org/10.1111/j.1439-0310.1997.tb00133.x>

Hopkin, S. P., & Martin, M. H. (1985). Assimilation of zinc, cadmium, lead, copper, and iron by the spider *Dysdera crocata*, a predator of woodlice. Bulletin of Environmental Contamination and Toxicology, 34(1), 183‑187. <https://doi.org/10.1007/BF01609722>

Horel, A., & Gundermann, J. L. (1992). Egg sac guarding by the funnel-web spider *Coelotes terrestris*: Function and development. Behavioural Processes, 27, 58‑94. <https://doi.org/10.1016/0376-6357(92)90018-9>

Horváth, R., Lengyel, S., Szinetár, C., & Honti, S. (2002). The effect of exposition time and temperature on spiders (Araneae) overwintering on the bark of black pine (*Pinus nigra*). In *European Arachnology*, Samu F. & Szinetár, C. (eds), pp. 95-102.

Huber, B. A. (1995). Genital morphology and copulatory mechanics in *Anyphaena accentuata* (Anyphaenidae) and *Clubiona pallidula* (Clubionidae: Araneae). Journal of Zoology, 235(4), 689‑702. <https://doi.org/10.1111/j.1469-7998.1995.tb01778.x>

Ingle, K., Kaur, H., Gallé-Szpisjak, N., Bürgés, J., Szabó, Á., & Gallé, R. (2020). Winter-Active Spider Fauna is Affected by Plantation Forest Type. Environmental Entomology, 49(3), 601‑606. <https://doi.org/10.1093/ee/nvaa025>

Kaston, B. J. (1976). Supplement to the Spiders of Connecticut. The Journal of Arachnology, 4(1), 1‑72.

Kim, K. W. (2000). Dispersal behaviour in a subsocial spider: Group conflict and the effect of food availability. Behavioral Ecology and Sociobiology, 48(3), 182‑187. <https://doi.org/10.1007/s002650000216>

Kim, K. W., & Horel, A. (1998). Matriphagy in the Spider *Amaurobius ferox* (Araneidae, Amaurobiidae): An Example of Mother‐Offspring Interactions. Ethology, 104(12), 1021‑1037. <https://doi.org/10.1111/j.1439-0310.1998.tb00050.x>

Kim, K. W., Roland, C., & Horel, A. (2000). Functional Value of Matriphagy in the Spider *Amaurobius ferox*. Ethology, 106(8), 729‑742. <https://doi.org/10.1046/j.1439-0310.2000.00585.x>

Koomen, P. (1998). Winter activity of *Anyphaena accentuata* (Walckenaer, 1802) (Araneae: Anyphaenidae). Proceedings of the 17^th^ European Colloquium of Arachnology, Edinburgh, Scotland.

Korenko, S., Pekár, S., & Honěk, A. (2010). Predation activity of two winter-active spiders (Araneae: Anyphaenidae, Philodromidae). Journal of Thermal Biology, 35(2), 112‑116. <https://doi.org/10.1016/j.jtherbio.2009.12.004>

Korenko, S., Sykora, J., Cernecka, L., Gajdos, P., Purgat, P., Cernecky, J., Holy, K., Heneberg, P., & Agnarsson, I. (2022). Elevation gradient affects the distribution and host utilisation of *Zatypota anomala* (Hymenoptera, Ichneumonidae) associated with mesh web weaving spiders (Araneae, Dictynidae). Journal of Hymenoptera Research, 93, 89-100. <https://doi.org/10.3897/jhr.93.91513>

Krafft, B., Horel, A., & Julita, J.-M. (1986). Influence of Food-Supply on the Duration of the Gregarious Phase of a Maternal-Social Spider, *Coelotes-terrestris* (Araneae, Agelenidae). Journal of Arachnology, 14(2), 219‑226.

Kronestedt, T. (1990). Separation of two species standing as *Alopecosa aculeata* (Clerck) by morphological, behavioural and ecological characters, with remarks on related species in the *pulverulenta* group (Araneae, Lycosidae). Zoologica Scripta, 19(2), 203‑225. <https://doi.org/10.1111/j.1463-6409.1990.tb00256.x>

Larsen, K. J., Brewer, S. R., & Taylor, D. H. (1994). Differential accumulation of heavy metals by web spiders and ground spiders in an old‐field. Environmental Toxicology and Chemistry, 13(3), 503‑508. <https://doi.org/10.1002/etc.5620130321>

Li, D., & Jackson, R. R. (1996). How temperature affects development and reproduction in spiders: A review. Journal of Thermal Biology, 21(4), 245‑274. <https://doi.org/10.1016/0306-4565(96)00009-5>

Mannu, R., Pantini, P., Sassu, A., & Verdinelli, M. (2020). A multidiversity approach to investigate the impact of mining exploitation on spider diversity in the abandoned mine district of Montevecchio-Ingurtosu (Sardinia, Italy). Environmental Science and Pollution Research, 27(26), 32615‑32627. <https://doi.org/10.1007/s11356-020-09553-y>

Mestre, L., Schirmel, J., Hetz, J., Kolb, S., Pfister, S. C., Amato, M., Sutter, L., Jeanneret, P., Albrecht, M., & Entling, M. H. (2018). Both woody and herbaceous semi-natural habitats are essential for spider overwintering in European farmland. Agriculture, Ecosystems & Environment, 267, 141‑146. <https://doi.org/10.1016/j.agee.2018.08.018>

Miliczky, E. R., Horton, D. R., & Calkins, C. O. (2008). Observations on phenology and overwintering of spiders associated with apple and pear orchards in south-central Washington. Journal of Arachnology, 36(3), 565‑573. <https://doi.org/10.1636/T07-29.1>

Nedvěd, O., Pekár, S., Bezděčka, P., Líznarová, E., Řezáč, M., Schmitt, M., & Sentenská, L. (2011). Ecology of Arachnida alien to Europe. BioControl, 56(4), 539‑550. <https://doi.org/10.1007/s10526-011-9385-3>

Nentwig, W. (1987). The prey of spiders. In Nentwig, W. (ed.) *Ecophysiology of Spiders*, pp 249-263, Springer, Berlin, Heidelberg. <https://doi.org/10.1007/978-3-642-71552-5_18>

Nyffeler, M. (2000). Do adult female lycosids feed during the period of maternal care? Bulletin of the British Arachnological Society, 11(9), 388-390.

Nyffeler, M., & Benz, G. (1988). Feeding ecology and predatory importance of wolf spiders (*Pardosa* spp.) (Araneae, Lycosidae) in winter wheat fields. Journal of Applied Entomology, 106(1‑5), 123‑134. <https://doi.org/10.1111/j.1439-0418.1988.tb00575.x>

Öberg, S. (2009). Influence of landscape structure and farming practice on body condition and fecundity of wolf spiders. Basic and Applied Ecology, 10(7), 614‑621. <https://doi.org/10.1016/j.baae.2009.03.005>

Pedersen, A. AA., & Loeschcke, V. (2001). Conservation genetics of peripheral populations of the mygalomorph spider *Atypus affinis* (Atypidae) in northern Europe. Molecular Ecology, 10(5), 1133‑1142. <https://doi.org/10.1046/j.1365-294X.2001.01266.x>

Pekár, S. (1999). Some observations on overwintering of spiders (Araneae) in two contrasting orchards in the Czech Republic. Agriculture, Ecosystems & Environment, 73(3), 205‑210. <https://doi.org/10.1016/S0167-8809(99)00052-3>

Pekár, S., & Beneš, J. (2008). Aged pesticide residues are detrimental to agrobiont spiders (Araneae). Journal of Applied Entomology, 132(8), 614‑622. <https://doi.org/10.1111/j.1439-0418.2008.01294.x>

Pekár, S., Coddington, J. A., & Blackledge, T. A. (2011). Evolution of stenophagy in spiders (Araneae): evidence based on the comparative analysis of spider diets. Evolution, 66(3), 776‑806. <https://doi.org/10.1111/j.1558-5646.2011.01471.x>

Pickavance, J. R. (2001). Life-cycles of four species of *Pardosa* (Araneae, Lycosidae) from the island of Newfoundland, Canada. Journal of Arachnology, 29(3), 367‑377. [https://doi.org/10.1636/0161-8202(2001)029[0367:LCOFSO]2.0.CO;2](https://doi.org/10.1636/0161-8202(2001)029%5b0367:LCOFSO%5d2.0.CO;2)

Piterkina, T. V., & Ovtsharenko, V. I. (2007). Interesting findings of spiders of the families Gnaphosidae and Liocranidae (Aranei) from the semidesert of the northern Caspian Lowland. Entomological Review, 87(9), 1317‑1324. <https://doi.org/10.1134/S0013873807090205>

Pollard, S. D., Jackson, R. R., Van Olphen, A., & Robertson, M. W. (1995). Does *Dysdera crocata* (Araneae Dysderidae) prefer woodlice as prey? Ethology Ecology & Evolution, 7(3), 271‑275. <https://doi.org/10.1080/08927014.1995.9522957>

Pywell, R. F., James, K. L., Herbert, I., Meek, W. R., Carvell, C., Bell, D., & Sparks, T. H. (2005). Determinants of overwintering habitat quality for beetles and spiders on arable farmland. Biological Conservation, 123(1), 79‑90. <https://doi.org/10.1016/j.biocon.2004.10.010>

Rabitsch, W. B. (1995). Metal accumulation in arthropods near a lead/zinc smelter in Arnoldstein, Austria. I. Environmental Pollution, 90(2), 221‑237. <https://doi.org/10.1016/0269-7491(94)00082-O>

Řezáč, M., Cardoso, P., & Řezáčová, V. (2023). Review of *Harpactea* ground-dwelling spiders (Araneae: Dysderidae) of Portugal. Zootaxa, 5263 (3), 335‑364. <https://doi.org/10.11646/zootaxa.5263.3.2>

Řezáč, M., Král, J., & Pekár, S. (2007). The Spider Genus *Dysdera* (Araneae, Dysderidae) in Central Europe: Revision and Natural History. The Journal of Arachnology, 35(3), 432-461. <https://www.jstor.org/stable/25067850>

Rickers, S., Langel, R., & Scheu, S. (2006). Stable isotope analyses document intraguild predation in wolf spiders (Araneae: Lycosidae) and underline beneficial effects of alternative prey and microhabitat structure on intraguild prey survival. Oikos, 114(3), 471‑478. <https://doi.org/10.1111/j.2006.0030-1299.14421.x>

Ruhland, F., Pétillon, J., & Trabalon, M. (2016). Physiological costs during the first maternal care in the wolf spider *Pardosa saltans* (Araneae, Lycosidae). Journal of Insect Physiology, 95, 42‑50. <https://doi.org/10.1016/j.jinsphys.2016.09.007>

Sas-Kovács, E. H., Urák, I., Cupsa, D., Sas-Kovács, I., Ferenti, S., & Rákosy, L. (2015). Wolf spider—Lycosidae—Assemblages of a decicuous forest in north-western Romania. Entomologia Generalis, 35(3), 199‑211. <https://doi.org/10.1127/entomologia/2015/0095>

Schmitz, A. (2016). Respiration in spiders (Araneae). Journal of Comparative Physiology B, 186(4), 403‑415. <https://doi.org/10.1007/s00360-016-0962-8>

Štempáková, K., & Hula, V. (2017). Overwintering of Spiders in Terrestrial Molluscs Shells in the Wider Area of Vranov nad Topľou (Slovakia). Acta Universitatis Agriculturae et Silviculturae Mendelianae Brunensis, 65(3), 947‑956. <https://doi.org/10.11118/actaun201765030947>

Thorbek, P., Sunderland, K. D., & Topping, C. J. (2004). Reproductive biology of agrobiont linyphiid spiders in relation to habitat, season and biocontrol potential. Biological Control, 30(2), 193‑202. <https://doi.org/10.1016/j.biocontrol.2003.10.004>

Ubick, D., & Vetter, R. S. (2005). A new species of *Apostenus* from California, with notes on the genus (Araneae, Liocranidae). Journal of Arachnology, 33(1), 63‑75. <https://doi.org/10.1636/H03-24>

Wang, X. P. (2002). A generic-level revision of the spider subfamily Coelotinae (Araneae, Amaurobiidae). Bulletin of the American Museum of Natural History, 269, 3‑150. <https://doi.org/10.1206/0003-0090(2002)269%3C0001:AGLROT%3E2.0.CO;2>

Whitney, T. E. D. (2014). Exploring the Links Between Seasonal Variation and Spider Foraging. Master Thesis, Entomology, College of Agriculture, 9, University of Kentucky.

Wilczek, G., Babczyńska, A., Augustyniak, M., & Migula, P. (2004). Relations between metals (Zn, Pb, Cd and Cu) and glutathione-dependent detoxifying enzymes in spiders from a heavy metal pollution gradient. Environmental Pollution, 132(3), 453‑461. <https://doi.org/10.1016/j.envpol.2004.05.011>

Yip, E. C., & Rayor, L. S. (2014). Maternal care and subsocial behaviour in spiders. Biological Reviews, 89(2), 427‑449. <https://doi.org/10.1111/brv.12060>

4. **WEBSITES**

Australian Museum. (s. d.). The Australian Museum. https://australian.museum/

British Arachnological Society. (s. d.). https://britishspiders.org.uk/

Joachim, J. (2014, juin 6). Les carnets nature de Jessica. https://jessica-joachim.com/

NatureWeb. (s. d.). Consulté 14 juin 2024. https://natureweb.com/

Oger, P. (s. d.). Les araignées de Belgique et de France. https://arachno.piwigo.com/

The Woodland Trust. (s. d.). Woodland Trust. https://www.woodlandtrust.org.uk/
